# Disulfide bonds are critical for stabilizing cell division, cell envelope biogenesis, and antibiotic resistance proteins in mycobacteria

**DOI:** 10.1128/mbio.01083-25

**Published:** 2025-07-31

**Authors:** Adrian Mejia-Santana, Rebecca Collins, Emma H. Doud, Cristina Landeta

**Affiliations:** 1 Department of Biology, Indiana University 1772, , Bloomington, Indiana, USA; 2 Biochemistry and Molecular Biology, Indiana University School of Medicine 12250, , Indianapolis, Indiana, USA; 3 Center for Proteome Analysis, Indiana University School of Medicine 12250, , Indianapolis, Indiana, USA; Washington University in St. Louis School of Medicine, St. Louis, Missouri, USA

**Keywords:** disulfide bonds, oxidative protein folding, DsbA, VKOR, substrates, essential proteins, PstP, PP2C, Ser/Thr phosphatase, EmbB, Rv2507, MmpS3, LamA, LpqW, MycP3, EccB3, AftD, AftB, mycobacteria, actinobacteria, mycomembrane

## Abstract

Mycobacteria, including *Mycobacterium tuberculosis*—the etiological agent of tuberculosis—possess a unique and impermeable cell envelope that is critical for survival and antibiotic resistance. The assembly and maintenance of this envelope depend on properly folded proteins, yet the role of disulfide bond formation in these processes remains poorly understood. Mycobacteria rely on two membrane enzymes, disulfide bond formation protein A (DsbA) and vitamin K epoxide reductase (VKOR), for introducing disulfide bonds into exported proteins. *In silico* studies predict that ~64% of exported proteins contain even numbers of cysteine residues and thence disulfide bonding; nevertheless, substrates of the DsbA-VKOR pathway remain largely unknown. Here, we demonstrate that DsbA and VKOR introduce disulfide bonds into substrate proteins and identify several essential proteins that depend on oxidative folding in the mycobacterial cell envelope. Using bioinformatics and cysteine profiling proteomics, we uncover numerous exported proteins that require disulfide bonds for stability. Cysteine derivatization in whole cells confirms that key proteins, including LamA (MmpS3), PstP, LpqW, and EmbB, rely on disulfide bonds for proper function. Furthermore, chemical inhibition of VKOR phenocopies *vkor* deletion, thus highlighting its essential role in maintaining mycomembrane integrity. These findings address a critical gap in understanding mycobacterial cell envelope biogenesis and underscore the DsbA-VKOR system as a promising target for disrupting cell envelope homeostasis in drug-resistant Mycobacteria.

**IMPORTANCE:**

This work addresses a major deficiency in understanding mycobacterial cell envelope processes and highlights the biological and clinical implications of oxidative protein folding in mycobacteria. This process, marked by the formation of disulfide bonds, is essential for the stability of exported proteins. While disulfide bond formation studies in Gram-negative bacteria suggested a similar role in mycobacteria, the underlying consequences of disulfide bonds remained unclear. Thus, we began investigating the diverse physiological functions dependent on disulfide bonds in Mycobacteria using a combination of bioinformatics, proteomics, and genetic and biochemical approaches. We identified hundreds of proteins affected by oxidative protein folding and validated essential substrates of this process. We show that disulfide bonds are not only crucial for the stability and function of key mycobacterial proteins but also represent a novel therapeutic target against antimicrobial resistance. Our findings underscore the potential of targeting disulfide bond formation to disrupt mycomembrane assembly, opening new avenues for antimycobacterial drug development.

## INTRODUCTION

*Mycobacterium tuberculosis*, the causative agent of tuberculosis, causes 1.5 million deaths worldwide every year, and the emergence of multidrug-resistant strains poses a serious threat to global health (1–3). In addition, nontuberculous mycobacteria (NTM), such as *M. abscessus* and *M. avium,* are an underrecognized source of lung disease, and the incidence of NTM disease has been surging worldwide, becoming an emerging public health problem (4). Mycobacteria have an intricate cell wall that represents a formidable barrier to drug entry and is a major contributor to resistance. Mycobacterial cell envelope is unusual in that it consists of mycobacterial peptidoglycan covalently linked to arabinogalactan, which is also covalently bound to mycolic acids (5). This complex structure is also known as the mycolyl-arabinogalactan-peptidoglycan complex (5). The bacterial cell envelope is the interface with the host and is critical to reacting to immune factors, tolerating antibiotic treatment, and adapting to the variable host environment (6). Thus, targeting the assembly and maintenance of the mycobacterial cell envelope represent a strategy against multidrug-resistant bacteria.

Oxidative protein folding is a promising novel target because disulfide bonds (DSBs) play a critical role in folding multiple exported proteins in the bacterial cell envelope. We and others have proposed targeting DSB-forming enzymes in pathogens as a new strategy to combat the antibiotic resistance crisis (7–10). Likewise, inhibition of DSB formation re-sensitizes multidrug-resistant clinical isolates of gram-negative bacteria, including *Escherichia coli, Klebsiella pneumoniae,* and *Stenotrophomonas maltophilia* to currently available antibiotics (11, 12).

**Fig 1:**
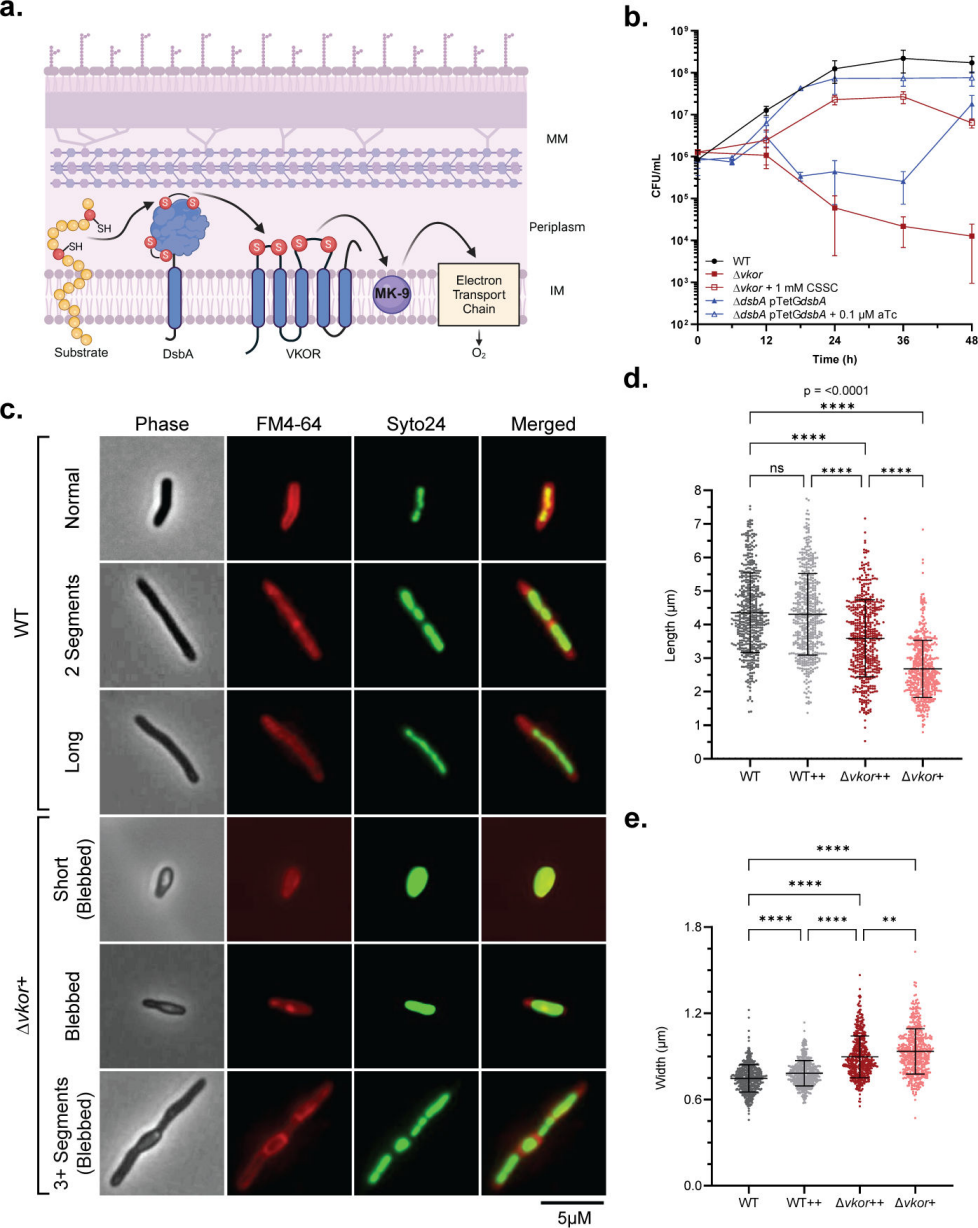
Lack of DSB formation causes growth and cell division defects in *M. smegmatis*. (a) DsbA oxidizes exported proteins and becomes reduced. VKOR regenerates reduced DsbA by transferring the electrons to menaquinone. Black arrows represent the flow of electrons, IM: inner membrane, MM: mycomembrane, MK-9: menaquinone-9. The schematic figure was created in BioRender. (b) *M. smegmatis* Δ*dsbA* and Δ*vkor* survival is affected in modified 7H9 broth. Δ*dsbA* was supplemented with 100 nM of aTc to induce Ms*dsbA* expression, and Δ*vkor* was supplemented with 1 mM cystine (CSSC). Data represent the average ±SD of at least three independent experiments. (c) WT and Δ*vkor* cells, supplemented with 1 mM (++) or 100 µM (+) cystine, were fluorescently stained with 50 nM Syto24 to stain nucleic acids and 0.6 µg/mL FM4-64 to stain the membrane. Representative images of the phenotypes are shown. (d and e) Cell dimensions were measured from samples obtained from at least three independent experiments using FIJI (https://fiji.sc/). Data represent the average ±SD. Cell counts included: WT (*n* = 495), WT++ (*n* = 505), Δ*vkor*++ (*n* = 498), Δ*vkor*+ (*n* = 500). Statistical tests were done using the Kruskal-Wallis multiple comparisons test. *P*-values are depicted in GP style: ≤ 0.0001 (****), 0.0002 (***), 0.021 (**), 0.0332 (*), and non-significant (ns).

In *E. coli,* DSB formation involves two enzymes. First, a periplasmic oxidoreductase, DsbA, enables appropriate folding of proteins by removing a pair of electrons (13). Then, the membrane protein DsbB regenerates DsbA’s activity by transferring electrons to quinones (14–16), either ubiquinone aerobically or menaquinone (aka Vitamin K2) anaerobically (17). Most proteobacteria have DsbAB homologs; conversely, actinobacteria, such as Mycobacteria, use an enzyme called Vitamin K epoxide reductase (VKOR) instead of DsbB (Fig. 1a) (18, 19). Bacterial VKOR is a homolog of the human VKORc1 enzyme (19, 20), yet bacterial and mammalian VKOR proteins display divergent mechanisms of electron transfer to quinones (21, 22).

While a detailed comparison of the mechanism of electron transfer between *M. tuberculosis* VKOR and *E. coli* DsbA revealed strong parallels to the *E. coli* DsbB and DsbA pair (23), the mycobacterial proteins have unique features. First, *M. tuberculosis* DsbA contains an additional pair of cysteines that form a DSB and is anchored to the plasma membrane by a single transmembrane (TM) segment (24–26). Second, VKOR contains five TM helices as opposed to four found in *E. coli* DsbB (27) or human VKORc1 (28). Finally, the *M. tuberculosis* predicted exported proteome identifies thousands of proteins that contain an even number of cysteine residues, suggesting that DSBs might be important for the folding and function of extracytoplasmic proteins (19). Nevertheless, substrates of the DsbA-VKOR pathway remain largely unknown.

In this work, we combined bioinformatics, cysteine-profiling proteomics, and genetic and biochemical approaches to demonstrate that DsbA and VKOR introduce DSBs into essential substrate proteins. We identified 787 proteins that were decreased in *M. smegmatis* Δ*vkor* grown under semi-permissive conditions, while 398 proteins increased. Furthermore, cysteine-specific enrichment proteomics revealed 745 oxidized cysteines corresponding to 381 exported proteins, which are potential substrates of DsbA. Using differential alkylation, we validated that *M. tuberculosis* LamA (MmpS3, Rv2198c), PstP (Rv0018c), LpqW (Rv1166), and EmbB (Rv3795) require one to two DSBs when expressed in *M. smegmatis,* a model mycobacterial species (29). We also demonstrated that the stability of these proteins is compromised in the absence of DSBs in Δ*vkor* and PstP cysteine mutants exhibit slow growth. Finally, we show as a proof of concept that chemical inhibition of VKOR phenocopies the Δ*vkor* mutant.

## RESULTS

### Disulfide bonds are important for cell division and septation in *M. smegmatis*

*M. tuberculosis* DsbA and VKOR essentiality was discovered through transposon mutagenesis (30). Similarly, *M. smegmatis* lacking the *vkor* gene could be generated when a small molecule oxidant, cystine (oxidized cysteine), is supplemented in the media (31) (Fig. 1b). When the Δ*vkor* is grown without cystine, there is a rapid decline in the population after 12 h (Fig. 1b), indicating the need for an oxidant to regenerate DsbA’s activity. Contrastingly, efforts to delete the *dsbA* homolog were unsuccessful with the addition of cystine (31). Most likely, cystine cannot oxidize secreted proteins directly but is more efficient in re-oxidizing DsbA in the Δ*vkor* (31). The deletion of *dsbA* is only possible in a merodiploid strain expressing *dsbA* from a regulatable promoter (31). This conditionally lethal mutant is unable to grow in the absence of the DsbA-inducer, anhydrotetracycline (aTc) (Fig. 1b). When this strain is grown without an inducer, however, suppressors arise after 36 h (Fig. 1b). These suppressors carry small insertions (7–22 bp) in *tetR* that cause frameshifts and premature stop codons, thereby disrupting *tetR*-mediated regulation. This type of mutation has previously been reported as high-frequency events (32).

We sought to determine the underlying mechanism DsbA-VKOR has on mycobacterial survival by first identifying morphological defects in *M. smegmatis* Δ*vkor* (Fig. 1c). When Δ*vkor* is grown under a limiting concentration of cystine (0.1 mM, Δ*vkor* +), the cells are smaller and wider compared to wild type (WT, Fig. 1d and e). These cells displayed membrane blebs indicative of cell wall defects and eventually, they lyse (Fig. 1c). While Δ*vkor* grows well under higher concentrations of cystine (1 mM, Δ*vkor* ++), there is still a population of smaller cells, indicating that cystine is not as effective at oxidizing DsbA as VKOR is (Fig. 1d). In addition, we observe that 63% of cells of the Δ*vkor* under low cystine display blebbed morphology, and 82% are undivided (Fig. S1a through c, Δ*vkor* +) compared to the 1% of blebbed and 27% of undivided cells observed in WT. The Δ*vkor* grown under semi-permissive conditions also displays 27% of cells with three or more segments compared to the 1% observed in WT (Fig. S1b). Altogether, these data suggest that a decrease in DSB formation causes a defect in cell division and septation.

### *In silico* analysis and cysteine profiling proteomics reveal potential substrates of mycobacterial DsbA

**Fig 2:**
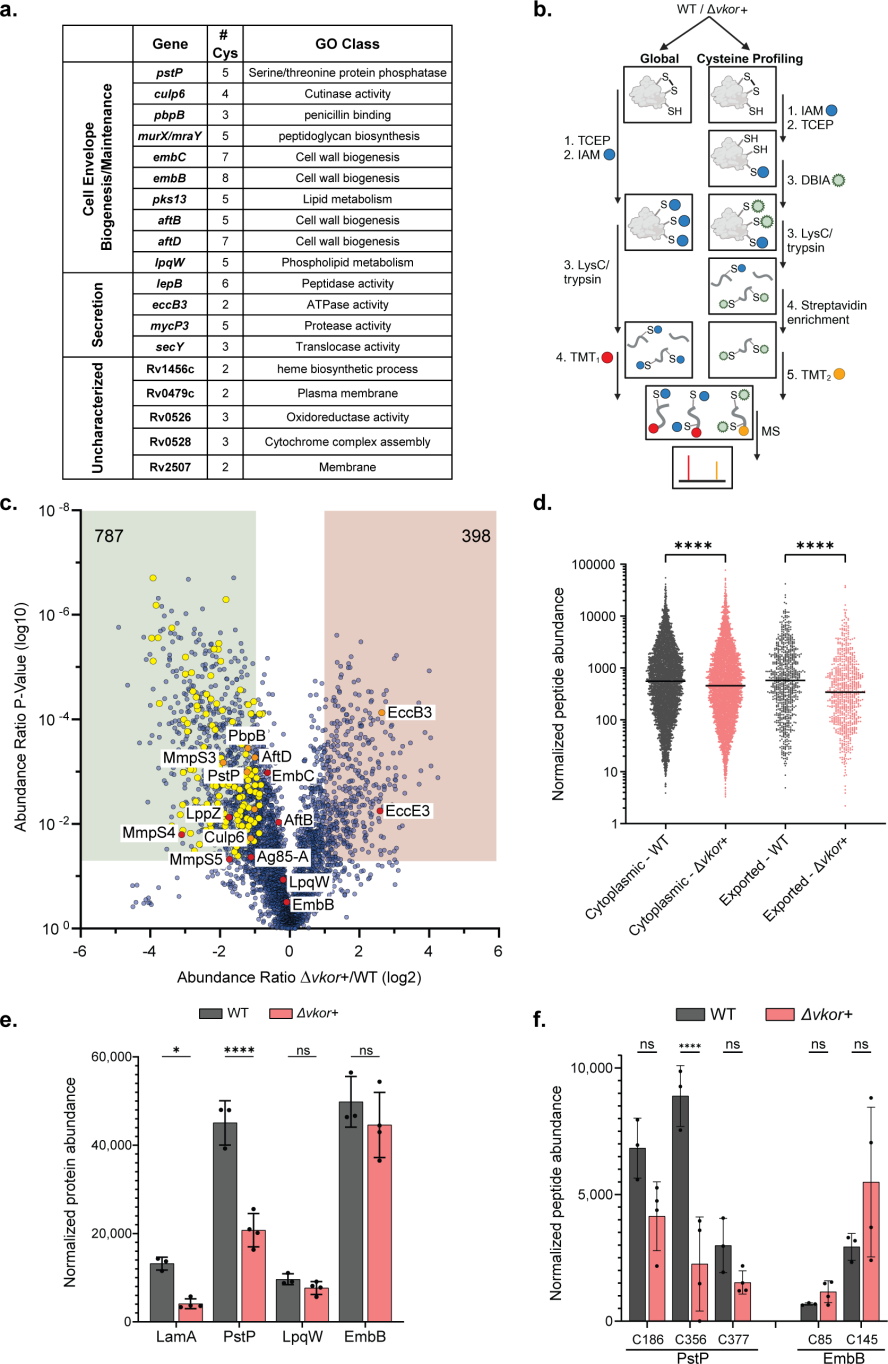
*In silico* and proteomic analyses reveal candidate DsbA substrates. (a) Bioinformatic analysis to mine essential exported genes identifies 19 candidate DsbA substrates (see Table S1). (b) Global and cysteine profiling proteomics approach to identify potential DsbA substrates (see text for further details). TCEP, tris(2-carboxyethyl)phosphine; IAM, iodoacetamide; DBIA, desthiobiotin-iodoacetamide; TMT, tandem mass tag; MS, mass spectrometry. The schematic figure was done with BioRender. (c) Volcano plot of protein abundance ratios of Δ*vkor*+ compared to WT (see Table S2). Decreased (green) or increased (pink) proteins with log2 ratios of ±1 and *P*-value ≤ 0.05 are highlighted. Red dots indicate proteins analyzed or discussed in this work (see Table S5), yellow dots indicate proteins for which cysteines were enriched and exported, and orange dots indicate essential proteins that overlap with *in silico* and proteomics approach (see Table S3). (d) Biotinylated-cysteine-containing peptides were sorted by the presence of TM segments or signal sequences and their normalized abundance was plotted. The data represent the median abundance. A statistical test was done using the Kruskal-Wallis multiple comparisons test. *P*-values are depicted in GP style: ≤ 0.0001 (****), 0.0002 (***), 0.021 (**), 0.0332 (*), and non-significant (ns). (e) The normalized abundance of four proteins of interest was detected by global proteomics. Data represent the average ±SD of at least three independent replicas. Statistical test was done using two-way ANOVA and Sidák’s multiple comparisons test. *P*-values are depicted in GP style: ≤ 0.0001 (****), 0.0002 (***), 0.021 (**), 0.0332 (*), and non-significant (ns). (f) Abundance of cysteine-containing peptides of two proteins of interest was detected by cysteine profiling proteomics. Data represent the average ±SD of at least three independent replicas. Statistical test was done using two-way ANOVA and Sidák’s multiple comparisons test. *P*-values are depicted in GP style: ≤ 0.0001 (****), 0.0002 (***), 0.021 (**), 0.0332 (*), and non-significant (ns).

Based on the morphologies seen in Δ*vkor*, we hypothesized that a protein involved in cell division and/or cell envelope integrity requiring DSBs is the reason for the DsbA-VKOR essentiality. Thus, we performed an *in silico* analysis using the previously identified 625 essential genes in *M. tuberculosis* (30). Since DSB formation occurs in exported proteins, we selected the essential proteins with a predicted signal sequence or TM segments. We narrowed down the list based on proteins experimentally confirmed to be exported (33) and harboring extracellular cysteines. Out of 625 proteins, 19 met these requirements and represent potential DsbA substrates (Fig. 2a). These proteins are conserved across five mycobacterial species as well as their cysteine residues (Table S1 and Fig. S2). The 19 candidates are mainly involved in cell envelope biogenesis and maintenance.

Parallel to the *in silico* analysis, we performed global and cysteine derivatization and enrichment proteomics using WT and Δ*vkor* (grown with 0.5 mM cystine) to identify oxidized cysteines. For this, we adapted a method previously developed to globally quantify modified cysteines in mouse tissues (34, 35). This approach relies on blocking reduced cysteines with an alkylating molecule, iodoacetamide (IAM), which binds irreversibly to free thiols (Fig. 2b). Oxidized (including disulfide-bonded) cysteines are then reduced with tris(2-carboxyethyl)phosphine (TCEP), and newly reduced cysteines are labeled with desthiobiotin-iodoacetamide (DBIA) (Fig. 2b). Proteins are then digested with trypsin, and desthiobiotinylated peptides are enriched using streptavidin beads for mass spectrometry analysis. This enrichment step is crucial for achieving broader coverage of the cysteine proteome (34). In parallel, the sample is also run for proteome-wide analysis to determine changes in protein abundance (Fig. 2b).

Through the global approach, we identified 4,021 unique proteins (Fig. 2c and Table S2a), of which 787 were significantly decreased in the Δ*vkor* + proteome (abundance ratio log2 ≤ −1 and *P*-value ≤ 0.05, Table S2b) while 398 proteins were significantly increased (abundance ratio log2 ≥ 1 and *P*-value ≤ 0.05, Table S2d). These changes in abundance could reflect cellular responses to envelope damage resulting from the absence of DSBs in certain proteins, as well as possible degradation, aggregation, or extraction biases inherent to the proteomics approach. We mined the protein data set for the presence of TM segments or signal sequences and found that 38% (298, Table S2c) of the decreased and 17% (66, Table S2e) of the increased proteins are predicted to be exported. We mapped protein-protein interactions within these two protein groups to determine their relationships, as some proteins may change in response to the absence of DSBs in other proteins. We found 22 clusters in the decreased and 12 in the increased abundance (Fig. S3). One key cluster includes cell division among the decreased proteins. This cluster has three essential proteins, including PstP, FtsW, and PknB (Table S3).

In addition, we identified 6,328 desthiobiotinylated (DB) cysteine-modified peptides through the enrichment approach (Table S2f). These peptides correspond to 5,607 unique cysteine residues and 2,545 unique proteins that experienced cysteine oxidation. From these, 87% (4,862 peptides) are cytoplasmic while 13% (745 peptides) belong to proteins with predicted TM or signal sequences (Table S2f). This reflects a greater-than-expected proportion of cytoplasmic proteins. While transient redox modifications in mycobacteria could partly explain this elevated enrichment, it is also possible that spontaneous oxidation or incomplete alkylation of cytoplasmic cysteines by IAM occurred during cell lysis (see Discussion). The median normalized abundance of DB-peptides in Δ*vkor* is 41% and 17% lower than WT in exported and cytoplasmic proteins, respectively (Fig. 2d). Although the reduction of some of these peptides is due to a decrease in the overall protein abundance (Table S2a). Lastly, the 745 DB-peptides correspond to 381 exported proteins that represent potential substrates of DsbA. Among these, 25 are essential, with some also found to be decreased, such as PstP, AftD, and LppZ, while others, including EccE3 and EccB3, were increased (Table S3).

Comparing the proteomics data with the *in silico* analysis, of the 19 predicted substrates, 2 were not detected in our data set—possibly because of technical limitations such as low expression levels, poor ionization efficiency, or limited tryptic peptide detectability. Among the remaining 17 proteins, 71% showed differential abundance and/or their cysteines were enriched (Table S3). The remaining 29% did not show significant changes in abundance, and their cysteines were not enriched. To determine the likelihood of DSBs in these 42 essential proteins identified by our *in silico* and proteomic approaches, we measured the distance between the alpha-carbon atoms (Cα-Cα) of the two cysteine residues closely located in the AlphaFold predicted structures (36) (Table S4). The distances between the Cα atoms for disulfide-bonded cysteines range between 3.0  Å and 7.5  Å (37). We found that 19 out of the 42 AlphaFold structures have a distance within this range, thus potentially linked by DSBs.

To validate potential DsbA-VKOR substrates found in our *in silico* and proteomics screens, we focused on four proteins shown to be crucial for cell division and antibiotic resistance in *M. tuberculosis*; two of these appeared in our proteomics approach and two in our bioinformatic analysis. First, we selected MmpS3 or LamA (for loss of asymmetry mutant A), which along with other MmpS homologs (MmpS4 and MmpS5) was significantly less abundant (Fig. 2c through e); and the bioinformatic analysis also identified an MmpS homolog, an essential protein of unknown function, Rv2507 (Fig. 2a). Mycobacterial MmpS proteins are usually not essential for survival (38), and they all harbor a conserved pair of cysteines (Fig. S4). LamA has a critical role in inhibiting cell wall synthesis at the new poles (39)—creating the characteristic asymmetric growth of mycobacteria that leads to heterogeneity in the cell population, and consequently impacts antibiotic resistance. Second, PstP is the only serine/threonine phosphatase in *M. tuberculosis* involved in growth rate, cell length, cell division, and cell wall metabolism (40–43). PstP appeared decreased in Δ*vkor* (Fig. 2e), its first three cysteines were enriched in the cysteine profiling, indicating oxidation, and the peptide with C356 was less enriched in Δ*vkor* + compared to WT (Fig. 2f and Table S2f). Third, LpqW, a lipoprotein that regulates phosphatidylinositol mannosides, lipomannan, and lipoarabinomannan, is required for mycomembrane biogenesis and thus an intrinsic contributor of antibiotic resistance (44, 45). LpqW is a candidate of the *in silico* analysis but shows no decrease in the proteome-wide analysis (Fig. 2e) and none of its cysteines were enriched. Fourth, EmbB, an arabinosyltransferase that catalyzes branching of arabinogalactan required for cell envelope biogenesis (46) and one of the targets of ethambutol, a first-line antituberculosis drug (47). EmbB appeared in the *in silico* search but showed no decrease in the global proteomics (Fig. 2e). The cysteines of EmbB were found in the oxidized cysteine enrichment in both WT and Δ*vkor* (Fig. 2f).

### *M. tuberculosis* LamA, PstP, LpqW, and EmbB require DsbA-VKOR for protein stability

**Fig 3:**
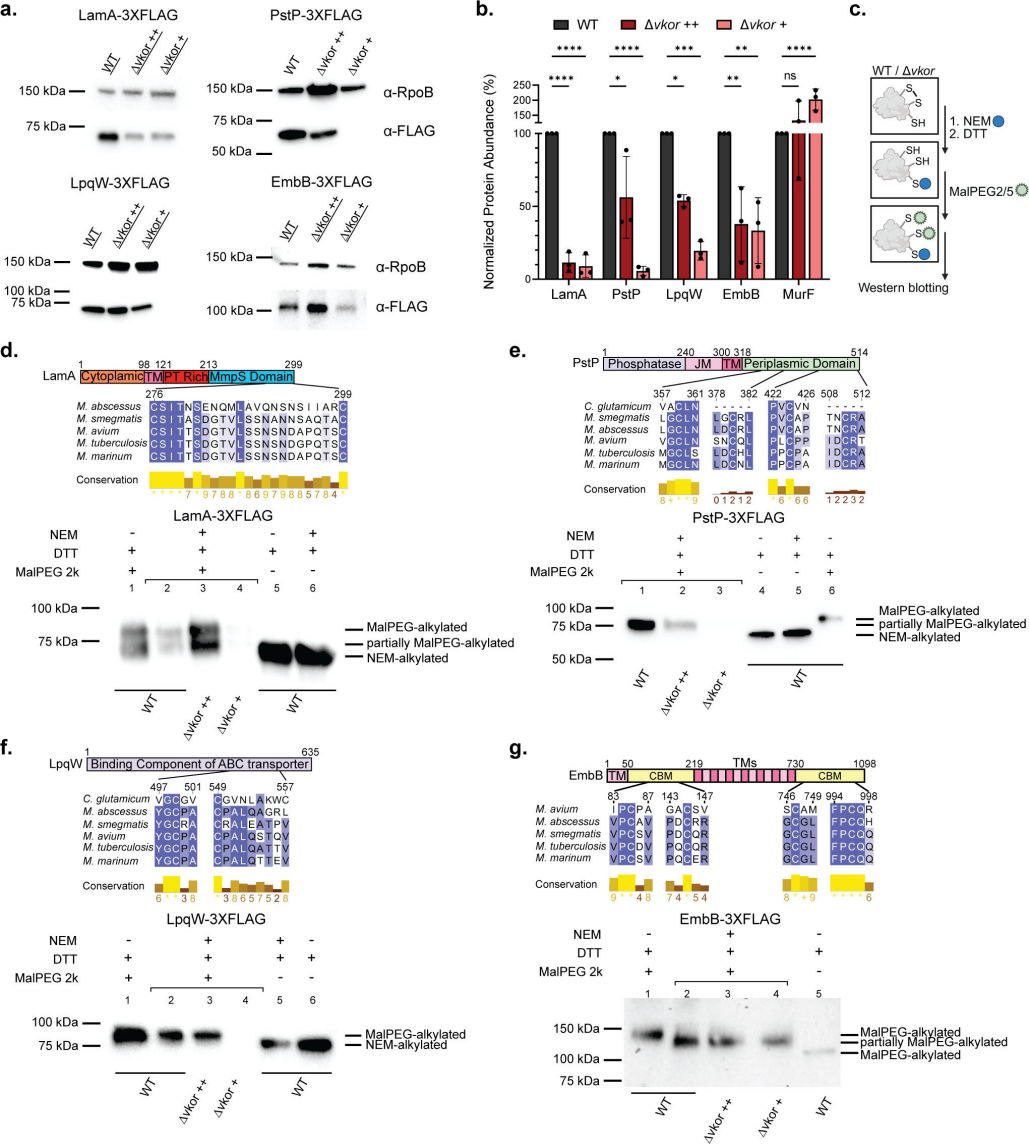
*M. tuberculosis* LamA, PstP, LpqW, and EmbB are substrates of DsbA/VKOR. (a) *Mt*LamA, *Mt*PstP, *Mt*LpqW, and *Mt*EmbB require DSBs for stability. *M. tuberculosis* proteins were fused to a 3X-FLAG tag at their carboxy termini and expressed in *M. smegmatis* WT and Δ*vkor* supplemented with 1 mM (++) or 0.4–0.5 mM cystine (+). Cells were grown at 37°C in the presence of 2.5 nM (EmbB) or 200 nM aTc for 36 h (PstP and LpqW), or 200 nM aTc for 18 h (LamA). Proteins were precipitated from cell lysates, quantified, and reduced before being separated by SDS-PAGE. Representative images are shown. (b) Protein abundance was determined by band density using α-RpoB as a loading control. Data represent average ±SD of three independent experiments. A statistical test was done using two-way ANOVA and Dunnett’s multiple comparisons test. *P*-values are depicted in GP style: ≤ 0.0001 (****), 0.0002 (***), 0.021 (**), 0.0332 (*), and non-significant (ns). (c) *In vivo* differential alkylation diagram (see text for further details). DTT, dithiothreitol; NEM, N-ethylmaleimide; MalPEG, α-[3-(3-Maleimido-1-oxopropyl)amino]propyl-ω-methoxy, polyoxyethylene, 2-5 kDa. (d) *Mt*LamA harbors one DSB. Top, multiple sequence alignment was done using Clustal Omega (48) and Jalview to visualize it (https://www.jalview.org/). Bottom, *M. smegmatis* WT and Δ*vkor* expressing *Mt*LamA were grown and induced as indicated in a. Experimental protein samples (indicated with a bracket) were differentially alkylated by treating them with 20 mM NEM to block free thiols. Disulfide-bonded cysteines were then reduced with 100 mM DTT, and new thiols were alkylated with 12.5 mM MalPEG2k. Controls were treated with 100 mM DTT and then alkylated with either 20 mM NEM or 12.5 mM MalPEG2k. Δ*vkor* samples were loaded in excess to be able to observe alkylated bands. Western blotting using α-FLAG antibody was used to detect LamA. The immunoblot is a representative image of at least three independent experiments. (e) *Mt*PstP harbors two DSBs. Similar to d. (f) *Mt*LpqW harbors one DSB. Similar to d. (g) *Mt*EmbB harbors two DSBs. Similar to d.

To determine whether these selected proteins require folding through DsbA-VKOR, we fused them to a 3X-FLAG tag at the carboxy terminus and ectopically expressed them in both *M. smegmatis* WT and Δ*vkor* mutant supplemented with permissive (Δ*vkor++*) and semi-permissive (Δ*vkor+*) concentrations of cystine. We found that *Mt*LamA is 91% less abundant in the *vkor* mutant grown under semi-permissive conditions (0.5 mM cystine) relative to WT (Fig. 3ab). Even under permissive growth (1 mM cystine), the same decrease in abundance, 88.6%, is observed. Similarly, *Mt*PstP is 43.8% lower in Δ*vkor* grown with 1 mM cystine and 94.3% lower grown with 0.4 mM cystine (Fig. 3ab). *Mt*LpqW displays a 46% and 80.5% reduction in the Δ*vkor* grown with high and low cystine, respectively (Fig. 3ab). As for *Mt*EmbB, we observe 62.1% and 66.7% reduction in the *vkor* grown under 1 mM and 0.5 mM cystine, respectively (Fig. 3ab). Importantly, protein degradation was not observed in the Δ*vkor* mutant, when expressing a cytoplasmic protein involved in peptidoglycan synthesis, *Mt*MurF (Fig. 3ab). Thus, these results suggest that *Mt*LamA, *Mt*PstP, *Mt*LpqW, and *Mt*EmbB may be misfolded and degraded due to the limited amount of DSBs in Δ*vkor*.

### *M. tuberculosis* LamA, PstP, LpqW, and EmbB harbor disulfide bonds

To investigate whether these proteins harbor DSBs when the DsbA-VKOR system is intact, we performed differential alkylation in whole cells (*in vivo*) to derivatize the reduced cysteines (Fig. 3c). This method consists of precipitating proteins from cell lysates and blocking irreversibly all free thiols with a light alkylating agent, NEM (N-ethylmaleimide, 0.125 kDa). Disulfide-bonded cysteines are then reduced with dithiothreitol (DTT), and all nascent free thiols are labeled with a second heavy alkylating agent, MalPEG2k (α-[3-(3-Maleimido-1-oxopropyl)amino]propyl-ω-methoxy, polyoxyethylene, 2 kDa). Thus, the molecular weight of the proteins with DSBs would show an increase in size of 2 kDa per cysteine, while non-disulfide bonded cysteines would have a marginal size change that can be identified via SDS-PAGE and western blotting. The advantage of this approach is that disulfide-bonded cysteines in a given protein display an increase in molecular weight as opposed to no shift in conventional alkylation labeling with only one heavy alkylating molecule.

*Mt*LamA is predicted to have a single TM helix and a periplasmic MmpS domain harboring the two conserved cysteines (Fig. S5a). *In vivo* differential alkylation revealed that *Mt*LamA, of a predicted size of 33.8 kDa, runs in a dimeric state of approx. ~68 kDa (Fig. 3d, lanes 5 and 6) and accumulates in an oxidized state (+4 kDa) in WT and Δ*vkor*, albeit at lower abundance in the mutant (~72 kDa. Fig. 3d, lane 2–4). A partially oxidized state with one cysteine labeled is also observed for WT and the Δ*vkor* mutant (Fig. 3d, lanes 2–4). This state could be an intermediate with a cysteine inaccessible to MalPEG2k due to steric hindrance, since it is also observed in the alkylated control rather than a DsbA-LamA intermediate. We used a heavier alkylating agent (MalPEG5k) to corroborate this finding and observed a similar result (Fig. S6a). Here, we also observed that Δ*vkor* with high and low cystine concentration has a small amount of the reduced form (Fig. S6a, lane 1 vs. 2–3), consistent with the reduced form of *Mt*LamA being unstable and degraded. Collectively, these results suggest the presence of one DSB in *Mt*LamA.

Similarly, we determined the presence of DSBs in *Mt*PstP. Its topology prediction indicates that one cysteine is located in the cytoplasm near one of the catalytic residues (D191) and four cysteines are located in the periplasm (Fig. S5b). *In vivo* differential alkylation indicates that *Mt*PstP is accumulated in a partially oxidized state in WT (Fig. 3e, lanes 1; Fig. S6b, lane 2). This state indicates that one cysteine is in a reduced state, hence alkylated with the light agent (+0.125 kDa), while the other four cysteines were labeled with the heavy agent (+8 kDa) after being reduced. Thus, *Mt*PstP with a predicted molecular weight of ~56.6 kDa (Fig. 3e, lane 4; Fig. S6b, lane 1) migrates slightly faster (~64.6 kDa, Fig. 3e, lane 1; Fig. S6b, lane 2) than the control with five labeled cysteines (~66.6 kDa. Fig. 3e, lane 6; Fig. S6b, lane 3). The partially oxidized state is also seen in the Δ*vkor,* although at lower abundance, dependent on cystine concentration (Fig. 3e, lane 2-3). No reduced form (~57.22 kDa) can be observed in the Δ*vkor*. We performed *in vivo* alkylation to corroborate this finding labeling only with MalPEG2k. We observe that *Mt*PstP displays a small shift of ~2 kDa (Fig. S6c, lane 1) above the non-alkylated control (Fig. S6c, lane 4), while the fully labeled control (Fig. S6c, lane 7) has a ~10 kDa shift as expected, thus indicating only one cysteine of PstP is reduced. Altogether, these results suggest the presence of two DSBs in *Mt*PstP.

Subsequently, *Mt*LpqW contains five cysteines, but two of these are located within the signal sequence and one belongs to the conserved lipobox motif (49) (Fig. S5c). Therefore, only two cysteines are potentially forming a DSB. Both *in vivo* differential alkylation with MalPEG2k and MalPEG5k show that LpqW (~66 kDa) is present in an oxidized state in WT and Δ*vkor* under high cystine concentration (Fig. 3f, lanes 2 and 3; Fig. S6d, lanes 2 and 3), and less of this form is observed in Δ*vkor* with low cystine, thus suggesting the presence of one DSB.

Lastly, *Mt*EmbB (~121 kDa) harbors fifteen TM segments and eight cysteines but only four of these are periplasmic (Fig. S5d). *In vivo* differential alkylation with MalPEG2k revealed a partial shift indicating a partially oxidized state (Fig. 3g, lane 1 vs 2). The half shift suggests that half of its cysteines are disulfide-bonded, indicating that *Mt*EmbB contains two DSBs. This partially oxidized form is also observed in Δ*vkor,* albeit in less abundance, with low cystine. Similarly to the other substrates, no reduced form can be detected (Fig. 3g, lane 2 vs 3–4).

In sum, we present evidence that *Mt*LamA, *Mt*PstP, *Mt*LpqW, and *Mt*EmbB harbor one, two, one, and two DSBs, respectively, in *M. smegmatis,* and the folded form of these proteins decreases in the *vkor* mutant in a dose-dependent manner to cystine concentration.

### *M. tuberculosis* PstP requires two consecutive and essential disulfide bonds

**Fig 4:**
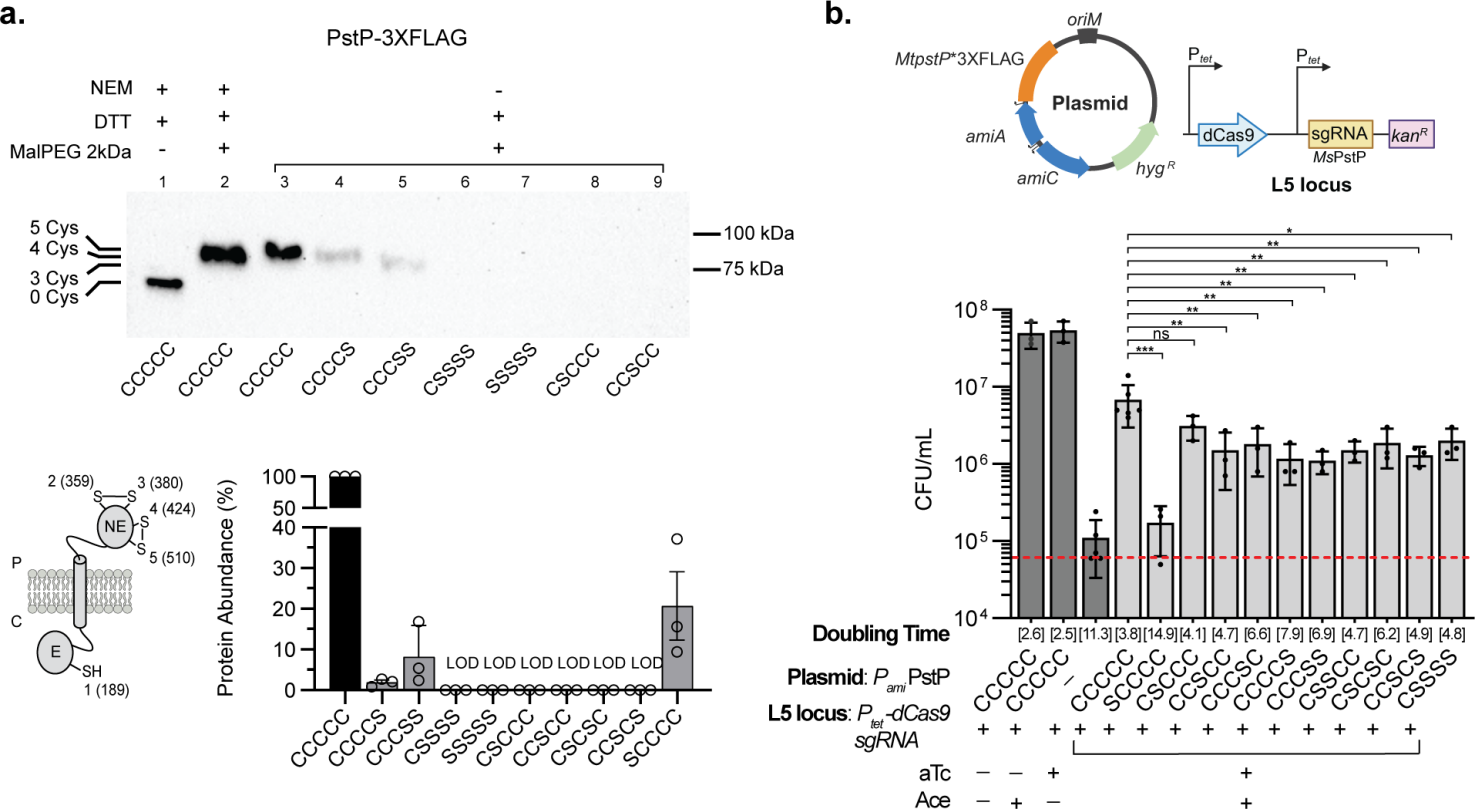
*M. tuberculosis* PstP harbors two essential consecutive DSBs. (a) The first DSB between Cys359 and Cys380 of PstP provides more protein stability than the DSB between Cys424 and Cys510. Cells were grown at 37°C for 36 h in the presence of 200 nM aTc. Proteins were precipitated, reduced with 100 mM DTT, and alkylated with 12.5 mM MalPEG2k. Controls were either reduced or differentially alkylated as indicated in Fig. 3. Immunoblot is a representative image of three independent experiments. Protein abundance was determined using reduced samples, and α-RpoB was used as a loading control. Data represent the average ±SD of three independent experiments. LOD: below the limit of detection. Cysteines are indicated in order: C189, C359, C380, C424, and C510. C, cytoplasm, P, periplasm. The schematic figure was created in BioRender. (b) DSBs in PstP are required for normal growth. *M. smegmatis pstP* was silenced using CRISPRi, while an ectopic copy of *M. tuberculosis pstP*, either WT or cysteine mutants, was used to rescue the knockdown growth. Cells were inoculated to an OD_600_ of 0.01 (CFU/mL indicated as red dotted line, see Source Data) in 7H9 broth supplemented with 400 nM anhydrotetracycline (aTc) and 25 µM of acetamide (Ace), and incubated at 37°C for 24 h to enumerate bacteria. Statistical tests were done using a one-way ANOVA multiple comparisons test. *P*-values are depicted in GP style: ≤ 0.0001 (****), 0.0002 (***), 0.021 (**), 0.0332 (*), and non-significant (ns). The schematic figure was created in BioRender.

Proteins containing two DSBs, such as *Mt*PstP and *Mt*EmbB, have two rearrangement options; either cysteines are disulfide bonded consecutively as they appear after being translocated to the periplasm (50) or they are rearranged by an isomerase between nonconsecutive cysteines after being oxidized by DsbA (51). We selected *Mt*PstP to determine the consecutiveness of the two DSBs, given the size, multiple TMs, and cysteines in *Mt*EmbB. To do this, we generated cysteine to serine changes in *Mt*PstP and compared their protein abundance when alkylated. We observed that when cysteines 4 and 5 are mutated, there is a 91.8% decrease in protein compared to WT (Fig. 4a, lane 4 and 5), while serine substitutions of cysteines 2 and 3 were below the limit of detection (Fig. 4a, lane 8 and 9). Noteworthily, the first cysteine (C189) mutant, which is located in the cytoplasm, also displays a 79.3% decrease, perhaps due to its proximity to the catalytic site (Fig. 4a). The catalytic site of PstP consists of five aspartates, one serine, and the backbone carbonyl of a glycine residue (Fig. S5b), which together form a binding pocket for three octahedrally coordinated Mn^2+^ ions and eight water molecules (52). The third Mn^2+^ ion—absent in the human PP2C phosphatase—is coordinated by D118, S160, D191, and two equatorial water molecules (52). These Mn^2+^ ions share an equatorial hydroxide ion or water molecule that acts as the nucleophile in a concerted hydrolytic attack on the substrate’s phosphoryl group (52). Altogether, our data suggest that *Mt*PstP harbors two consecutive DSBs, and the first DSB between C359 and C380 provides slightly more stability than the DSB between C424 and C510. It is possible that the C189S substitution, located near D191, disrupts the solvation or coordination of one of the Mn^2+^ ions, thereby impairing the structural integrity of the active site.

To test the role of each of the two DSBs in *Mt*PstP, we generated a knockdown using a previously developed CRISPRi system for mycobacteria (53). We inserted *tet*-regulated dCas9 and sgRNA targeting *MspstP* at the L5 site and complemented it with an ectopic copy of *MtpstP* under the control of the acetamidase promoter (P*_ami_*). Induction of CRISPRi with 400 nM aTc silences chromosomal *MspstP*, which prevents viability (Fig. 4b, aTc) and causes a characteristic blebbing phenotype in cells (41) (Fig. S7a). Growth and morphology can then be restored by expressing *MtpstP* with 25 µM acetamide (Fig. 4b; Fig. S7a). We replaced the *MtpstP* copy with cysteine mutant alleles to determine the essentiality of the two DSBs. Even though all periplasmic cysteine mutants were unstable as shown above (Fig. S7b), they affected the growth rate of the *pstP* knockdown to varying degrees (Fig. 4b, average doubling time 4.4 h vs. 3.8 h for WT). This slower growth rate is comparable to the decrease observed when the non-essential periplasmic domain is deleted (40). By contrast, the cytoplasmic cysteine mutant had a more severe impact on viability (doubling time 14.9 h), possibly due to its proximity to the catalytic domain, as discussed above. Interestingly, mutants disrupting the second DSB exhibited a slower growth rate (average doubling time 7.2 h) than those lacking the first DSB (average doubling time 4.4 h) despite having opposite effects on protein degradation (Fig. 4b). These differences are not due to resistance to CRIPSRi, as all mutants showed reduced viability in the absence of acetamide (Fig. S7c). It is possible that cysteine mutants disrupting the first DSB are cleaved off, leaving the cytoplasmic domain partially functional, whereas the cysteines disrupting the second DSB are not fully separated from the misfolded portion of the protein and therefore less functional. Notably, the characteristic blebbing phenotype observed in *pstP* mutants is slightly more pronounced in strains lacking the first (21%) or both (28%) rather than the second DSB (16%) (Fig. S7a), indicating that the growth rate and morphology phenotypes are distinct. Altogether, our data suggest that both consecutive DSBs in *Mt*PstP are required for normal growth.

### Inhibition of mycobacterial VKOR phenocopies the Δ*vkor* mutant

**Fig 5:**
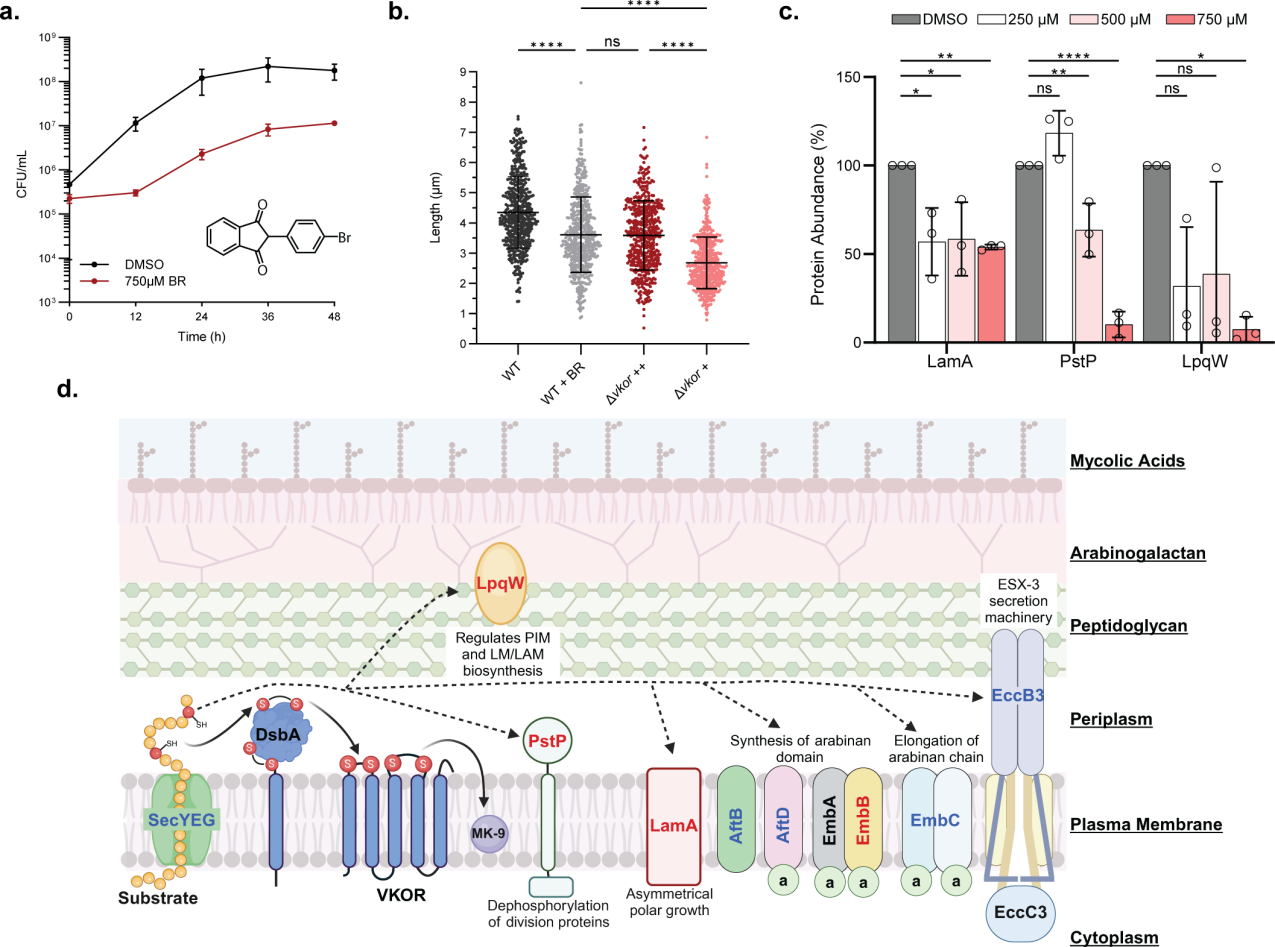
Targeting oxidative protein folding simultaneously affects multiple essential cell envelope proteins. (a) *M. smegmatis* WT treated with DMSO or 750 µM bromindione (BR) was grown in modified 7H9 broth at 37°C. Data represent the average ±SD of at least three independent experiments. (b) *M. smegmatis* WT treated with DMSO or 750 µM BR was grown in modified 7H9 broth at 37°C for 36 h. Cells were fluorescently stained with 50 nM Syto24 to stain nucleic acids and 0.6 µg/mL FM4-64 to stain the membrane. Cell dimensions were measured using FIJI (https://fiji.sc/). Data represent the average ±SD. Cell counts included: WT (*n* = 495), WT + BR (*n* = 490), Δ*vkor*++ (*n* = 498), and Δ*vkor*+ (*n* = 500). Statistical tests were done using the Kruskal-Wallis multiple comparisons test. *P*-values are depicted in GP style: ≤ 0.0001 (****), 0.0002 (***), 0.021 (**), 0.0332 (*), and non-significant (ns). (c) Essential proteins are unstable when *M. smegmatis* is grown with a VKOR inhibitor (BR). *M. smegmatis* expressing FLAG-tagged proteins was grown with different concentrations of BR at 37°C for 24 (*Mt*LamA) or 36 h (*Mt*PstP and *Mt*LpqW). Proteins were precipitated from cell lysates and reduced before being separated by SDS-PAGE. Protein abundance was determined using α-RpoB as a loading control. Values represent the average ±SD of three independent experiments. Statistical tests were done using one-way ANOVA and Dunnett’s multiple comparisons test. *P*-values are depicted in GP style: ≤ 0.0001 (****), 0.0002 (***), 0.021 (**), 0.0332 (*), and non-significant (ns). (d) DSBs are present in proteins involved in *M. tuberculosis* cell envelope biogenesis and cell division (see text for further details). Proteins in red text were experimentally demonstrated to have DSBs in this work, while proteins in blue text are predicted substrates identified in this work and structural studies (see Table S5). Solid black arrows represent the flow of electrons, and dotted black arrows indicate the substrates of the DsbA/VKOR pathway. MK-9, menaquinone; PIM, phosphatidylinositol mannosides; LM, lipomannan, LAM, lipoarabinomannan; a, acyl-carrier protein; ESX-3, specialized secretion system. The schematic figure was created in BioRender.

We determined, as a proof of principle, whether inhibition of VKOR in *M. smegmatis* can have the same effect as Δ*vkor* on essential proteins. We used bromindione (BR), an oral anticoagulant, which is a modest but, so far, one of our best inhibitors of mycobacterial VKOR (22, 54). When *M. smegmatis* is grown in 750 µM BR, the minimal inhibitory concentration (MIC) displays slower growth compared to the WT control (Fig. 5a), and this condition phenocopies the Δ*vkor* grown on high cystine (Fig. 1b). The cell length and morphology (Fig. 5b, Fig. S8a and b) of BR-treated cells also resemble the Δ*vkor* grown on high cystine concentration. Furthermore, cells have a 91% reduction of disulfide-bonded *Mt*PstP and *Mt*LpqW, while a 46.1% decrease in *Mt*LamA when incubated with 750 µM BR compared to the vehicle control (Fig. 5c). The decrease in protein stability is dependent on bromindione concentration (Fig. 5c), and the effect of the highest BR concentration in *Mt*PstP and *Mt*LpqW levels is comparable to the Δ*vkor* grown on low cystine concentration. Thus, VKOR inhibition affects, to varying degrees, the essential substrates.

## DISCUSSION

We sought to identify essential client proteins of oxidative protein folding driven by DsbA-VKOR in mycobacteria (Fig. 5d and Table S5). We found that 298 out of 4,021 identified proteins are predicted to be exported and are decreased in abundance when Δ*vkor* is grown under semipermissive conditions. In addition, cysteine profiling proteomics identified 381 exported proteins with oxidized cysteines, from which 132 proteins overlap with proteins that showed decreased abundance. Together, these findings suggest that ~14% of the proteome consists of potential substrates of DsbA-VKOR. We showed that the inner membrane proteins EmbB, PstP, and LamA, together with the lipoprotein LpqW, require one or two intramolecular DSB(s) for stability. We provide evidence that the two DSBs in *M. tuberculosis* PstP are consecutive and required for normal growth.

Our *in silico* and proteomic analyses identified 19 essential proteins containing oxidized cysteines, a markedly higher proportion than in *E. coli*, where only three essential proteins depend on DSBs (55). This substantial difference suggests that the essentiality of the DsbA-VKOR pathway in mycobacteria may stem from the simultaneous degradation of several misfolded essential proteins, ultimately leading to membrane instability and cell lysis. Of the four proteins examined in this study—PstP, LpqW, EmbB, and LamA—three are essential for mycobacterial viability. Although LamA is not essential, it plays a crucial role in establishing cell asymmetry and heterogeneity (39). PstP contains an essential cytoplasmic catalytic domain and a nonessential periplasmic domain with an as-yet-unknown function in both *M. smegmatis* and *M. tuberculosis* (40). LpqW, a regulator of glycolipid branching, is essential in *M. tuberculosis* and required for normal growth in *M. smegmatis* (30, 38, 44). The arabinosyltransferase EmbB, which catalyzes arabinogalactan branching (46), is essential in *M. tuberculosis* (30, 38). In *M. smegmatis*, deletion of *embB* results in slow growth, shortened cell length, membrane blebbing, and increased permeability to hydrophobic drugs and detergents (56).

In our data set, we enriched 5,607 cysteines out of the 16,816 cysteines in the *M. smegmatis* proteome. Of these, 4,862 cytoplasmic (28.9%), while 745 periplasmic (4.4%). This represents a higher-than-anticipated proportion of cytoplasmic proteins (32% of the total proteome) compared to the 15%–20% reported for eukaryotic cells (57). While transient redox modifications in mycobacteria could be one of the reasons for this higher enrichment, it is also possible that spontaneous oxidation or incomplete IAM blocking during the cell lysis may have occurred. To note, the cytoplasmic cysteine of PstP was enriched in the streptavidin enrichment. However, when we performed sequential alkylation, this cysteine was found reduced. This may suggest that IAM blocking was incomplete for some cysteines in the enrichment approach. Thus, the extent and significance of cytoplasmic cysteine oxidation should be evaluated in future studies by reversing the order of the alkylating agents, DBIA and IAM.

In addition to essential disulfide-bonded substrates, a protein part of the type VII secretion system, EspA, has been found to require an intermolecular DSB (58). *M. tuberculosis* EspA harbors only one cysteine that allows the formation of a homodimer in both *M. tuberculosis* and *M. smegmatis* (58). EspA was found in *M. tuberculosis* cell pellets (58), so it may be translocated to the periplasm, where DsbA introduces the DSB to then be secreted as a homodimer. In agreement with our findings, there are examples of *M. tuberculosis* exported proteins containing disulfide bonds in their crystal structures (Table S5). In these studies, heterologous expression of antigen 85A (59), LpqW (60), Culp6 (61), EmbC (62), MycP1 (63), MycP3 (64), PbpB (65), AftD (66), and MmpS5 (67) indicates the presence of DSB(s) when expressed in *E. coli* as well as EccB3 (68) when expressed in *M. smegmatis*. While the presence of DSBs based on structure indicates this structural component, determining whether DSBs are present in the native organism is more challenging for some proteins. For instance, MycP3 was correctly produced in *E. coli’*s periplasm using a fusion to the *pelB* signal sequence and purified without reducing agents (64). However, LpqW, Culp6, MycP1, PbpB, and EmbC’s carboxy-terminal domain were produced without the signal peptide or TM segment, hence in the reductive environment of *E. coli’*s cytoplasm (60–63, 65), and LpqW was reduced during purification (60, 69). So, most likely, the DSBs are formed by air oxidation (70). On the contrary, the cryo-EM structure of *Mt*EmbB does not indicate the presence of DSBs even though the cysteines are close to each other in the structure (71, 72). These studies do not report the use of a reducing molecule during protein purification from *M. smegmatis* (72) or *E. coli* (71). Possibly, radiation damage occurred since cysteines and DSBs are particularly susceptible to high doses of electron beams used in cryo-EM studies (73–75), or perhaps the amount of protein expressed is too high for DsbA to oxidize/recognize it, and air oxidation did not introduce the DSBs. Thus, determining DSBs in a protein requires expression in the right compartment of the native or closely related host as well as *in vivo* cysteine labeling to ascertain their presence.

Instability due to the absence of DSBs and consequent protein degradation was observed for all four proteins—LamA, PstP, LpqW, and EmbB—when overexpressed in the Δ*vkor* strain. However, in our proteomic analysis, there was no detectable difference in the abundance of LpqW and EmbB. This discrepancy between the degradation observed during overexpression and the lack of differential detection in proteomics may reflect technical limitations inherent to proteomics approaches. Specifically, both LpqW and EmbB were identified at low abundance (7.3rd percentile for LpqW and 22.6th for EmbB), and with limited peptide coverage, which likely reduced the statistical power to confidently detect changes in stability.

In addition, in the differential alkylation experiments, the reduced forms of these proteins were not detected—except for LamA—further suggesting that the reduced forms are unstable and possibly degraded. On the other hand, the cysteine mutant of EspA is slightly degraded but overall more stable (58), perhaps because EspA’s DSB is intermolecular, rather than intramolecular, and hence not needed for protein stability. Something to note is that the extent of proteolysis varied depending on the protein. For instance, LamA is completely degraded in the Δ*vkor* supplemented with high vs. low cystine. However, there were more oxidized forms of PstP, LpqW, and EmbB when Δ*vkor* was grown with high rather than low cystine; either DsbA may be able to recognize these as a priority, or the protease senses more than LamA misfolding over the others. Degradation of misfolded proteins in the periplasm of *E. coli* is one of the functions of the protease DegP (76). In mycobacteria, there are three orthologs of HtrA (for high temperature requirement) protease, including the secreted protease *htrA3*/*pepA* and the membrane-anchored proteases *htrA* and *htrA2*/*pepD* (77). Of these, only HtrA is essential for survival, and the amidase Ami3 is one recently identified substrate (77). None of these appeared increased in our proteomic analysis; however, components of the proteasome, including ClpX, Mpa, and Bpa, showed higher peptide abundance. Determining which periplasmic protease(s) degrade misfolded proteins in DSB formation mutants will require further investigation.

Proteins that harbor two DSBs can acquire two configurations, either consecutive or non-consecutive. For PstP, the differences in stability of the cysteine mutants suggest that these are consecutive DSBs. As for EmbB, the consecutive cysteines appear close in the structure, so they are most likely consecutive DSBs. The introduction of non-consecutive DSBs in *E. coli* requires an isomerization pathway directed by DsbCD proteins (51, 78). Previous *in vitro* and structural studies have identified *M. tuberculosis* isomerase-like proteins and a probable regenerating partner (79, 80). However, it is unknown whether isomerization takes place in mycobacteria and what proteins would require isomerization of DSBs in the mycobacterial cell envelope.

The essential catalytic domain of PstP lies in the cytoplasm (41). By contrast, the periplasmic domain is dispensable, and its function remains to be identified. We found that the lack of cysteines or the DSBs in the non-essential domain of PstP causes protein instability and degradation. This parallels *E. coli* FtsN, which also contains a DSB in its non-essential SPOR (sporulation-related repeat) domain (81). However, the extent of degradation in FtsN cysteine mutants does not allow *E. coli* survival (81), whereas the degradation observed in PstP cysteine mutants allows partial survival. These differences may be due to the essential domain of FtsN located on the periplasmic side, whereas the essential domain of PstP lies in the cytoplasm.

We point out that the presence of DSBs in several exported proteins offers a valuable opportunity to investigate the mechanisms of protein folding and mycomembrane assembly in mycobacteria, paralleling studies in *E. coli* outer membrane biogenesis (55, 82–84). Finally, our findings underscore that disrupting DSB formation in the mycobacterial cell envelope could simultaneously compromise several essential proteins, as illustrated by the impact of a weak mycobacterial VKOR inhibitor. VKOR proteins are attractive drug targets, with human VKORc1 serving as a precedent. Human VKORc1 catalyzes the regeneration of active vitamin K to support blood coagulation and is the target of oral vitamin K antagonists such as warfarin, which is widely used to treat and prevent thromboembolic diseases (20). Warfarin interferes with cofactor binding by forming two hydrogen bonds with conserved VKORc1 residues (28). Bacterial and mammalian VKOR proteins have distinct cofactor binding strategies (21, 22). Building on this concept, our group is actively developing mycobacterial VKOR inhibitors that selectively target the bacterial enzyme without affecting human VKORc1 (10, 22, 54). We propose that effective DsbA-VKOR inhibitors would exert enhanced bactericidal effect and may also sensitize mycobacteria to existing antibiotics that target the cell envelope, representing a promising therapeutic strategy.

## MATERIALS AND METHODS

### Bacterial strain and growth conditions

Bacterial strains used in this study are listed in Table S6. *E. coli* DH10β cells (New England Biolabs) were used for plasmid construction and grown at 37°C in NZ broth (shaking in an Innova incubator at 250 rpm) or agar plates with suitable antibiotics. *M. smegmatis* mc^2^155 was grown at 37°C on NZ agar plates or modified Middlebrook 7H9 broth supplemented with 0.5% bovine serum albumin, 0.2% D-glucose, 0.2% glycerol, 0.05% Tween 80, and additional phosphates including 19.38 mM K_2_HPO_4_, 10.12 mM KH_2_PO_4_ to buffer the acidity of L-cystine. The antibiotic concentrations used were hygromycin 200 µg/mL for *E*. *coli* and 100 µg/mL for *M. smegmatis*; kanamycin 40 µg/mL for *E*. *coli* and 25 µg/mL for *M. smegmatis. M. smegmatis* Δ*vkor* strains were grown in the presence of 1.5 mM L-cystine (Sigma) unless stated otherwise.

### Strain construction

Primers used in this study are listed in Table S7. To express *M. tuberculosis* proteins into *M. smegmatis,* genes were amplified from the *M. tuberculosis* H37Rv genome and cloned under the *tet* promoter of the pTetG plasmid. The vector was generated by amplifying the pTetG plasmid using primers PR173 and PR151 to then insert the *M. tuberculosis pstP* gene amplified with primers PR174 and PR154. The fragments were then ligated using NEBuilder HiFi Assembly (New England Biolabs) to obtain the PL105 plasmid. The right insertion was sequenced with primers PR172 and PR230. Construction of subsequent expression plasmids was made by amplifying the vector with primers PR173 and PR151 using PL105 as a template. The gene inserts were amplified from the *M. tuberculosis* genome with primer pairs: PR231 and PR249 for PL148, PR372 and PR373 for PL220, and primers PR330 and PR312 for PL265.

To have an alternative inducible system, we constructed a plasmid inducible with acetamide. For this, plasmid PL283 was constructed by removing the TetR of PL105 using primers PR476 and PR477 and re-ligating the product with a KLD mix (New England Biolabs). The intermediate vector was then amplified using PR474 with PR475. The minimal acetamidase regulon (85) was then amplified from pJV126 using primers PR472 and PR473 and ligated via NEBuilder HiFi Assembly (New England Biolabs) to the intermediate vector to obtain PL286. The plasmid was confirmed by sequencing with primers PR478, PR479, and PR480.

PstP cysteines were mutagenized by site-directed mutagenesis (KLD mix, New England Biolabs) using PL105 as a template using primers PR222 and PR223 to construct PL275 and PL299; PR224 and PR225 to make PL276 and PL300; PR278 and PR229 for PL209 and PL302; and PR289 with PR290 for PL291 and PL298. The cumulative cysteine knockout mutants were generated through site-directed mutagenesis reactions using the single cysteine mutant plasmid as the template until all five cysteines of PstP were knocked out.

To silence Ms*pstP* expression, we used a dCas9_Sth1_ system optimized for *M. smegmatis* (53). The system uses a vector that expresses *Streptococcus thermophilus dcas*9 and the sgRNA scaffold under the control of the *tet* promoter (TetR). The PAM sequence CGAGAAG with a score of 1 (a.k.a. high silencing strength) was used (53). The ∼20 bp *pstP* targeting region (amino acid 5) was cloned into the sgRNA scaffold using aligned primers PR496 and PR497 (heating equimolar concentrations at 95°C and cooling at 25°C) and assembled with BsmBI-digested vector (Golden Gate Assembly, New England Biolabs) using T4 DNA ligase, T4 Polynucleotide Kinase (New England Biolabs). The resulting plasmid, PL282, was sequenced with primer PR291 and transformed into *M. smegmatis,* selecting for kanamycin resistance to obtain strain LL464. Induction of *dcas9* with 400 nM aTc caused poor *M. smegmatis* growth.

All plasmids were electroporated into *M. smegmatis* by first growing cells to an OD_600_ of 0.8–1.0 in NZ broth. Cells were then washed and resuspended in cold 10% glycerol. An aliquot of 100 µL electrocompetent cells was transformed with ~500 ng of DNA and electroporated in 2 mm electroporation cuvettes using Gene Pulser II with Pulse Controller Plus and Capacitance Extender Plus accessories (Bio-Rad) with settings 2.5 kV, 1,000 Ω, and 25 µF. Transformants were recovered using 1 mL of modified-7H9 broth and incubated shaking for 2–4 h at 37°C. Cells were then plated on selective NZ agar plates and/or L-cystine. *M. smegmatis* was independently transformed with plasmids PL105, PL146, PL148, PL220, PL265, PL209, PL243, PL249, PL252, PL258, PL275, PL276, PL288, PL289, PL290, PL291, and PL282 to generate the following strains: LL148, LL189, LL191, LL343, LL419, LL398, LL399, LL400, LL401, LL402, LL423, LL424, LL468, LL469, LL470, LL471, and LL464, respectively. Similarly, *M. smegmatis* Δ*vkor* was independently transformed with plasmids PL105, PL146, PL148, PL220, and PL265 to generate strains LL163, LL194, LL196, LL344, and LL420, respectively. Finally, LL464 was independently transformed with the following plasmids: PL286, PL298, PL299, PL300, PL301, PL302, PL303, PL304, PL305, PL306, and PL307 to generate the following strains: LL466, LL514, LL515, LL516, LL517, LL518, LL519, LL525, LL526, LL527, and LL528, respectively.

### Growth assay

*M. smegmatis* WT, Δ*vkor*, and Δ*dsbA* pTetG*dsbA* were grown in 7H9 broth, supplemented with either 1 mM L-cystine for Δ*vkor* or 0.1 µM of aTc (Sigma) for Δ*dsbA*. Cultures were diluted to an OD_600_ of 0.02 (~10^5^ cells) in fresh 7H9 media supplemented with 1 mM L-cystine or 0.1 µM of aTc when appropriate. Cultures were incubated at 37°C and shaken at 225 rpm for 48 h. Aliquots were taken at each 12 h timepoint, serially diluted with phosphate-buffered saline (PBS) + 0.05% Tween 80, and plated on NZ media with supplements where necessary. Plates were incubated at 37°C for 72 h to enumerate colonies.

For *MspstP* silencing experiments, overnight cultures were diluted to an OD_600_ of 0.01 in 6 mL of 7H9 broth supplemented with 400 nM aTc and/or 25 µM acetamide. Cells were grown at 37°C for 24 h. Aliquots were serially diluted with PBS + 0.05% Tween 80 and plated on NZ media. Plates were incubated at 37°C for 72 h to enumerate colonies.

### Fluorescence microscopy analysis

*M. smegmatis* WT and Δ*vkor* were grown in 7H9 broth at 37°C for 48 h. Overnight cultures were then diluted to an OD_600_ of 0.01 in 6 mL of 7H9 broth, and Δ*vkor* was supplemented with either 1 mM (permissive, ++) or 100 µM (semi-permissive, +). Cells were grown at 37°C to an OD_600_ < 0.3, washed, and resuspended in PBS with 0.05% Tween 80. For *MspstP* silencing experiments, overnight cultures were diluted to an OD_600_ of 0.01 in 6 mL of 7H9 broth supplemented with 400 nM aTc and/or 25 µM acetamide. Cells were grown at 37°C for 24 h. Nucleic acid dye, SYTO24 (Invitrogen), and membrane dye FM4-64 (Invitrogen) were added to diluted cells at a final concentration of 50 nM and 0.6 µg/mL, respectively. Cells were then incubated for 15 min in the dark and spotted onto a 2% agarose pad. A glass coverslip was laid on top of the agarose and imaged on a Nikon Ti2E microscope equipped with a Plan Apo 100×/1.4NA phase contrast oil objective and an sCMOS camera. Several pictures of at least three independent experiments were taken and processed using FIJI (86) to determine cell dimensions.

### Cysteine profiling proteomics

This protocol was adapted from reference (34). Briefly, three *M. smegmatis* WT and four Δ*vkor* biological replicates were grown in 7H9 broth at 37°C for 48 h. Cultures were then diluted in 200 mL (Δ*vkor*) or 50 mL (WT) of 7H9 to an OD_600_ of 0.02. The Δ*vkor* samples were supplemented with 0.5 mM cystine (Δ*vkor*+) and incubated for 43 h at 37°C and 250 rpm of orbital shaking. Cells were harvested and resuspended in lysis buffer containing 50 mM NH_4_HCO_3_, 10 mM MgCl_2_, 1 mM EDTA, 7 M Urea, 10 µM phenyl-methyl-sulfonyl fluoride (PMSF, Thermo Scientific), pH 7.4, and mechanically lysed using a Mini-Beadbeater (BioSpec). Beating cycles were performed five times for 30 seconds with 2 min ice incubation intervals. Samples were then centrifuged at 13,000 rpm for 10 min at 4°C and cell lysates were subjected to acid-quenching precipitation using 10% TCA (Sigma). Samples were then incubated on ice for 20 min. The protein precipitate was pelleted and washed with cold acetone. Protein pellets were dried and solubilized in 1 mL of labeling buffer (100 mM HEPES pH 8.5, containing 8 M urea, 2% SDS, and 1 mM EDTA). The total protein concentration was determined by Pierce BCA assay (Thermo Fisher Scientific) and aimed for 1,000 µg of total protein. The sample was then split into two tubes.

One half-sample “differentially alkylated” was resuspended in blocking buffer with 35 mM iodoacetamide (IAM, Sigma) for 2 h at 37°C in the dark to block all unmodified cysteine residues, while the other half sample “global” was treated with 10 mM TCEP for 1 h at room temperature to reduce all cysteines. Proteins in both half-samples were precipitated with 10% TCA and resuspended in 500 µL of labeling buffer. The differentially alkylated samples were then reduced with 10 mM TCEP for 1 h at room temperature to reduce disulfide-bonded cysteines, while the global samples were treated with 35 mM IAM for 2 h at 37°C in the dark. The counter-alkylated samples were TCA precipitated and resuspended in 500 µL of labeling buffer with desthiobiotin iodoacetamide (DBIA; CAS No. 2924824-04-2, Santa Cruz Biotechnology). Both differentially alkylated and global samples were precipitated by methanol and chloroform. Protein pellets were resuspended in 500 µL of 100 mM HEPES pH 8.0 and digested with LysC and trypsin (Thermo Fisher Scientific) for 16 h at room temperature. Global peptides were flash-frozen until use, and differentially alkylated peptides were diluted in binding PBS buffer (0.1 M sodium phosphate, 0.15 M sodium chloride, pH 7.4). Pierce High-Capacity Streptavidin Agarose beads were equilibrated as per the manufacturer’s instructions (Thermo Fisher Scientific) and incubated with peptides for 4 h at room temperature. Beads were washed four times with 100 mM HEPES pH 8 and 0.05% NP-40, four times with 100 mM HEPES pH 8, and one time with high-purity water to then be resuspended in 50 µL 100 mM HEPES pH 8 and stored at 4°C until processing.

### Peptide purification and labeling

Peptide purification, mass spectrometry analysis, bioinformatics, and data evaluation for quantitative proteomics experiments were performed in collaboration with the Indiana University Proteomics Center for Proteome Analysis at the Indiana University School of Medicine, similarly to previously published protocols (87, 88). Following enrichment for DBIA-modified peptides, peptides were eluted from the beads with 3 × 100 µL washes of 80% acetonitrile, 0.1% formic acid (FA) for 20 min at room temperature, 10 min at room temperature, and 10 min at 72°C all at 600 rpm shaking. These elutions were combined and dried by speed vacuum. For global proteomics, approximately 100 µg of peptides was desalted on Waters Sep-Pak Vac cartridges (Waters Cat No: WAT054955) with a wash of 1 mL 0.1% TFA followed by elution in 3 × 0.2 mL of 70% acetonitrile 0.1% FA. Peptides were dried by speed vacuum and resuspended in 50 mM triethylammonium bicarbonate (TEAB, from 1 M stock). Each sample was then labeled for 2 hours at room temperature, with 0.5 mg of Tandem Mass Tag Pro reagent (manufacturer’s instructions, Thermo Fisher Scientific, TMTpro Isobaric Label Reagent Set; Cat No: 44520, lot no. XC34531). TMTpro labels used for global analysis included WT three replicas: 127N, 128N, and 129N and Δ*vkor* four replicas: 131N, 132N, 133N, and 134N, respectively. TMTpro labels used for cysteine profiling analysis included WT three replicas: 126C, 127C, and 128C and Δ*vkor* four replicas: 129C, 131C, 132C, and 133C, respectively. Samples were checked to ensure > 90% labeling efficiency and then quenched with 0.3% hydroxylamine (vol/vol) at room temperature for 15 minutes. Labeled peptides were then mixed and dried by speed vacuum.

### High pH basic fractionation

Half of the combined global sample and all of the DBIA-peptide sample were resuspended in 0.5% TFA and fractionated on a Waters Sep-Pak Vac cartridge (Waters Cat No: WAT054955) with a 1 mL wash of water, 1 mL wash of 5% acetonitrile, 0.1% triethylamine (TEA) followed by elution for the global sample in 8 fractions of 12.5%, 15%, 17.5%, 20%, 22.5%, 25%, 30%, and 70% acetonitrile, all with 0.1% TEA) and three fractions for the DBIA peptide sample of 12.5%, 22.5%, and 70% acetonitrile with TEA.

### Nano-LC-MS/MS

Mass spectrometry was performed utilizing an EASY-nLC 1200 HPLC system (SCR: 014993, Thermo Fisher Scientific) coupled to an Eclipse mass spectrometer with FAIMSpro interface (Thermo Fisher Scientific). Each multiplex was run on a 25 cm Aurora Ultimate TS column (Ion Opticks Cat No: AUR3-25075C18) in a 50°C column oven with a 180 minute gradient. For each fraction, 2% of the sample was loaded and run at 350 nL/min with a gradient of 8%–38% B over 98 minutes; 30%–80% B over 10 min; held at 80% for 2 minutes; and dropping from 80% to 4% B over the final 5 min (Mobile phases A: 0.1% FA, water; B: 0.1% FA, 80% Acetonitrile; Thermo Fisher Scientific Cat No: LS122500). The mass spectrometer was operated in positive ion mode, default charge state of 2, advanced peak determination on, and lock mass of 445.12003. Three FAIMS CVs were utilized (−45 CV, −55 CV, and −65CV and a technical replicate with −40 CV, −50 CV, and −60 CV) each with a cycle time of 1 s and with identical MS and MS2 parameters. Precursor scans (m/z 400–1,600) were done with an orbitrap resolution of 120,000, RF lens% 30, 50 ms maximum inject time, standard automatic gain control (AGC) target, minimum MS2 intensity threshold of 2.5e4, MIPS mode to peptide, including charges of 2 to 6 for fragmentation with 60 sec dynamic exclusion shared across the cycles excluding isotopes. MS2 scans were performed with a quadrupole isolation window of 0.7 m/z, 34% HCD collision energy, 50,000 resolution, 200% AGC target, dynamic maximum IT, and fixed first mass of 100 m/z.

### Mass spectrometry data analysis

Resulting RAW files were analyzed in Proteome Discover 2.5.0.400 (Thermo Fisher Scientific [89]) with an *M. smegmatis* UniProt reference proteome FASTA (downloaded 080924, 6,600 sequences) plus common laboratory contaminants (73 sequences) and the HPV16E6 protein sequence. SEQUEST HT searches were conducted with full trypsin digest, two maximum number missed cleavages; precursor mass tolerance of 10 ppm; and a fragment mass tolerance of 0.02 Da. Static modifications used for the search were as follows: (i) TMTpro label on peptide N-termini and (ii) TMTpro label on lysine (K). Dynamic modifications used for the search were (i) carbamidomethylation on cysteine (C) residues, (ii) DBIA probe (+296.185) on cysteine, (iii) oxidation on methionine (M) residues, (iv) acetylation on protein N-termini, (v) methionine loss on protein N-termini, or (vi) acetylation with methionine loss on protein N-termini. A maximum of three dynamic modifications were allowed per peptide. Percolator false discovery rate was set to a strict setting of 0.01 and a relaxed setting of 0.05. IMP-ptm-RS node was used for all modification site localization scores. Values from both unique and razor peptides were used for quantification. In the consensus workflows, peptides were normalized by total peptide amount with no scaling. Unique and razor peptides were used, and all peptides were used for protein normalization and roll-up. Quantification methods utilized TMTpro isotopic impurity levels available from Thermo Fisher Scientific. Reporter ion quantification filters were set to an average S/N threshold of 5 and a co-isolation threshold of 30%. Resulting grouped abundance values for each sample type, abundance ratio values, and respective *P*-values (Protein Abundance based on ANOVA, individual protein based) from Proteome Discover were exported to Microsoft Excel. In the global analysis, 4,024 unique proteins were identified, as well as 39,789 peptide groups and 274,054 PSMs. Cysteine profiling analysis identified 13,157 peptides, of which 6,328 were DB-modified peptides (49% enrichment selectivity for cysteine-containing peptides). From these, 5,607 were unique cysteines that belonged to 2,546 unique proteins. Exported proteins containing either signal sequences or transmembrane segments were found using DeepTMHMM 1.0 (https://services.healthtech.dtu.dk/services/DeepTMHMM-1.0/) (90).

### *In vivo* differential alkylation

*M. smegmatis* WT and Δ*vkor* strains were grown in 7H9 broth at 37°C for 48 h. Cultures were then diluted in 7H9 to an OD_600_ of 0.02. The Δ*vkor* samples were supplemented with 1 mM (permissive, ++) or 0.4–0.5 mM (semi-permissive, +) cystine. Strains expressing FLAG-tagged proteins were induced with 2.5 nM (EmbB) or 0.2 µM aTc and grown at 37°C for 36 h (EmbB, PstP, and LpqW). LamA-harboring strains were incubated for only 18 h at 37°C with 0.2 µM aTc to prevent protein aggregates. Cells were harvested and resuspended in lysis buffer containing 50 mM NH_4_HCO_3_, 10 mM MgCl_2_, 1 mM EDTA, 7 M Urea, 10 µM phenyl-methyl-sulfonyl fluoride (PMSF, Thermo Fisher Scientific), pH 7.4, and mechanically lysed using a Mini-Beadbeater (BioSpec). Beating cycles were performed five times for 30 seconds with 2 min ice incubation intervals. Samples were then centrifuged at 13,000 rpm for 10 min, and cell lysates were subjected to acid-quenching precipitation using 10% trichloroacetic acid (TCA, Sigma). Samples were then incubated on ice for 30 min. The protein precipitate was pelleted and washed with cold acetone twice. Protein pellets were dried and solubilized in 100 mM Tris pH 8, containing 1% SDS. The total protein concentration was determined by Pierce BCA assay (Thermo Fisher Scientific) prior to the addition of reducing or alkylating agents.

For *in vivo* differential alkylation, the experimental samples were first treated with 20 mM NEM (N-ethylmaleimide, Sigma) and incubated at 37°C for an hour to block free thiols. M63 0.2% glucose media was added to bring the volume to 900 µL, and proteins were re-precipitated with 10% TCA followed by acetone washes. Precipitated proteins were then solubilized in 100 mM Tris pH 8 containing 1% SDS (and 1 mM EDTA for EmbB samples), and disulfide-bonded cysteines were reduced by incubating with 100 mM DTT (dithiothreitol, Sigma) for 30 minutes at room temperature. Samples then underwent another round of TCA precipitation to be subsequently alkylated with either 12.5 mM MalPEG2k (α-[3-(3-Maleimido-1-oxopropyl)amino]propyl-ω-methoxy, polyoxyethylene, NOF America Corporation) or MalPEG5k and incubated for an hour at 37°C. Controls included samples treated with 100 mM DTT and then alkylated with 20 mM NEM, 12.5 mM MalPEG2k, or MalPEG5k using the same conditions described above.

For *in vivo* alkylation, proteins were precipitated as mentioned above and treated with MalPEG2k for an hour at 37°C. Controls included samples treated with 100 mM DTT and then alkylated with either 20 mM NEM or 12.5 mM MalPEG2k using the same conditions described above.

### Western blot analysis and protein quantification

Protein samples were normalized to 2.5–30 µg of total protein, mixed with 5×-SDS reducing loading buffer (100 mM DTT helped to decrease the background), and boiled for 5 min at 100°C (except for EmbB samples, which were incubated at room temperature for 15 min) to proceed to SDS-PAGE. Bio-Rad Mini-PROTEAN TGX 4%–20% or 7.5% gels were run for 65 min at 150 V. Proteins were then transferred onto PVDF membrane (Millipore) using Trans-Blot SD Semi Dry Transfer Cell (Bio-Rad) for 50 min at 0.07A. Membranes were then blocked with TBS (Tris Buffered Saline) containing 5% milk and incubated with a 1:10,000 dilution of α-FLAG antibody (Sigma) overnight at 4°C. Similarly, 1:10,000 dilution of α-RpoB antibody (Invitrogen) was used as a loading control. After incubation with primary antibodies, the blots were washed three times with TBS containing 0.1% Tween 20 and incubated with corresponding secondary antibody, either a 1:50,000 dilution of α-Rabbit antibody (Santa Cruz Biotechnology) or a 1:25,000 dilution of α-Mouse antibody (Santa Cruz Biotechnology) for 1 h at room temperature. Chemiluminescent substrate (ECL, Bio-Rad) was used to detect proteins using ChemiDoc MP Imaging System (Bio-Rad). For *in vivo* differential alkylation, proteins were not normalized to load because *vkor* samples would show low signal; thus, these were loaded in fivefold to sixfold excess to be able to see alkylated bands. To determine protein abundance, proteins were treated with DTT only, separated, and transferred to membranes, which were cut to blot independently with the two antibodies. The adjusted total band volume was determined for FLAG and RpoB images using Image Lab 6.1 Software. The arbitrary units obtained for each band were then normalized to the volume obtained in the WT protein band. The relative band volume of FLAG was then divided by the relative band volume of RpoB to obtain the relative protein abundance. Anti-FLAG antibody was validated using a total protein extract of *M. smegmatis* without FLAG tag, there were no non-specific bands for α-FLAG raised in rabbit, while α-FLAG raised in mouse produced a non-specific band at ~50 kDa, which was indicated when appropriate.

### Drug assay

*M. smegmatis* overnight cultures were diluted to an OD_600_ of 0.02 in 6 mL of 7H9 broth supplemented with 750 µM brominedione (CAS1146-98-1, Santa Cruz Biotechnology) and incubated shaking at 225 rpm and 37°C for 48 h. Aliquots were taken every 12 h, serially diluted with PBS + 0.05% Tween 80, and plated on NZ media. Plates were incubated at 37°C for 72 h to enumerate colonies. At 36 h of growth, one aliquot was used to stain with SYTO24 and FM4-64 and analyzed by microscopy as described above.

*M. smegmatis* expressing FLAG-tagged proteins were inoculated in 7H9 broth to an OD_600_ of 0.02 in fresh 7H9 media containing 0.2 µM aTc and varying concentrations of brominedione (250, 500, and 750 µM) or DMSO (Sigma) as vehicle control. Cells were grown at 37°C for 36 h, or 24 h for *Mt*LamA to prevent protein aggregation. Proteins were precipitated and reduced to determine abundance using anti-FLAG antibody as indicated above.

### Bioinformatic analysis

*In silico* analysis was performed on all the previously identified essential genes in *M. tuberculosis* (30). GI accession numbers of 625 essential genes were first acquired through the Database of essential genes (DEG) database (http://origin.tubic.org/deg/public/index.php) and used to obtain the FASTA protein sequences using the National Center for Biotechnology Information (NCBI, https://www.ncbi.nlm.nih.gov/). To determine which proteins are predicted to be secreted, we used SignalP-5.0 (91) and TOPCONS (92), which predict the presence of signal sequences and TM segments, respectively. Out of the list of 625 proteins, 47 were predicted to have TM segments and 130 were predicted to be secreted. Proteins were further narrowed down by a more recent essentiality study (38) to 90 proteins. Through manual curation of TOPCONS plots, proteins predicted to harbor cysteine residues in the extracellular compartment were chosen from the pool, which resulted in a final list of 25 proteins as candidate DsbA substrates. Finally, a literature search was performed on those to disqualify proteins that were not experimentally demonstrated to be periplasmic (33), ending with a final list of 19 proteins (Table S1).

### Statistical analysis

Comparisons of the cell size mean between mutant and wild type with or without bromindione were done using the Kruskal-Wallis multiple comparisons test with GraphPad Prism version 10.0.0 (USA). Comparisons of the peptide abundance mean between mutant and wild type were done using non-paired Kruskal-Wallis with Dunn’s multiple comparison test using GraphPad Prism version 10.4.1. Comparisons between the normalized abundance of peptides of the wild-type control and Δ*vkor* were done using ordinary non-paired one-way ANOVA multiple comparisons, selecting for Sidáks test with GraphPad Prism version 10.5.0. Comparisons between protein abundance of the wild-type control and Δ*vkor* were done using ordinary non-paired two-way ANOVA multiple comparisons, selecting for Dunnett’s test with GraphPad Prism version 10.5.0. Comparisons between the survival mean of the wild type control vs. PstP cysteine mutants were done using ordinary non-paired one-way ANOVA multiple comparisons, selecting for Dunnett’s test with GraphPad Prism version 10.4.0. Significant differences were indicated in graphs using GP style: *P*-value ≤ 0.0001 (****), 0.0002 (***), 0.021 (**), and 0.0332 (*). Non-significant *P*-values (>0.1234) were indicated as ns. *P*-values for all statistical tests are provided in Source Data as Supplemental Information.

## Data Availability

Supplemental Information contains 8 supplementary figures, and 7 supplementary tables in a word document. Table S2 and the Source Data file ( Data Set S1) containing unprocessed images and the data/analyses used to generate graphs are provided as two separate Excel files. Raw and processed mass spectrometry data are uploaded to the MassIVE repository with accession MSV000096877 and ProteomeXchange PXD059898.

## SUPPLEMENTAL MATERIAL

Data Set S1 mbio.01083-25-s0001.xlsx Unprocessed images and the data/analyses used to generate graphs.

Supplemental Material mbio.01083-25-s0002.docx Figures S1 to S8 and Tables S1 to S7.

Table S2 mbio.01083-25-s0003.xlsx Proteomics raw data set.

ASM does not own the copyrights to Supplemental Material that may be linked to, or accessed through, an article. The authors have granted ASM a non-exclusive, world-wide license to publish the Supplemental Material files. Please contact the corresponding author directly for reuse.
