# Supplementary material for: Disulfide bonds are critical for stabilizing cell division, cell envelope biogenesis, and antibiotic resistance proteins in mycobacteria
Source: mBio. 2025 Jul 31;16(9):e01083-25. doi: 10.1128/mbio.01083-25 (PMC12421883; doi:10.1128/mbio.01083-25)
Supplement: Supplemental Material — Figures S1 to S8 and Tables S1 to S7. [file mbio.01083-25-s0002.docx]

**Supplementary Information**

**Disulfide bonds are critical for stabilizing cell division, cell envelope biogenesis and antibiotic resistance proteins in mycobacteria**

Running Title: Essential cell envelope proteins harbor disulfide bonds in Mycobacteria

Adrian Mejia-Santana^1^, Rebecca Collins^1^, Emma H. Doud^2,3^, and Cristina Landeta^1*^

Author’s affiliations:

^1^ Department of Biology. Indiana University. Bloomington, IN USA.

^2^ Biochemistry and Molecular Biology. Indiana University School of Medicine. Indianapolis, IN U.S.A.

^3^ Center for Proteome Analysis; Indiana University School of Medicine. Indianapolis, IN U.S.A.

^*^Correspondence should be addressed to Dr. Cristina Landeta: clandeta@iu.edu

**Keywords:** disulfide bonds, oxidative protein folding, DsbA, VKOR, substrates, essential proteins, PstP, PP2C, Ser/Thr phosphatase, EmbB, Rv2507, MmpS3, LamA, LpqW, MycP3, EccB3, AftD, AftB, mycobacteria, actinobacteria, mycomembrane.

**Supplementary Figures**

**
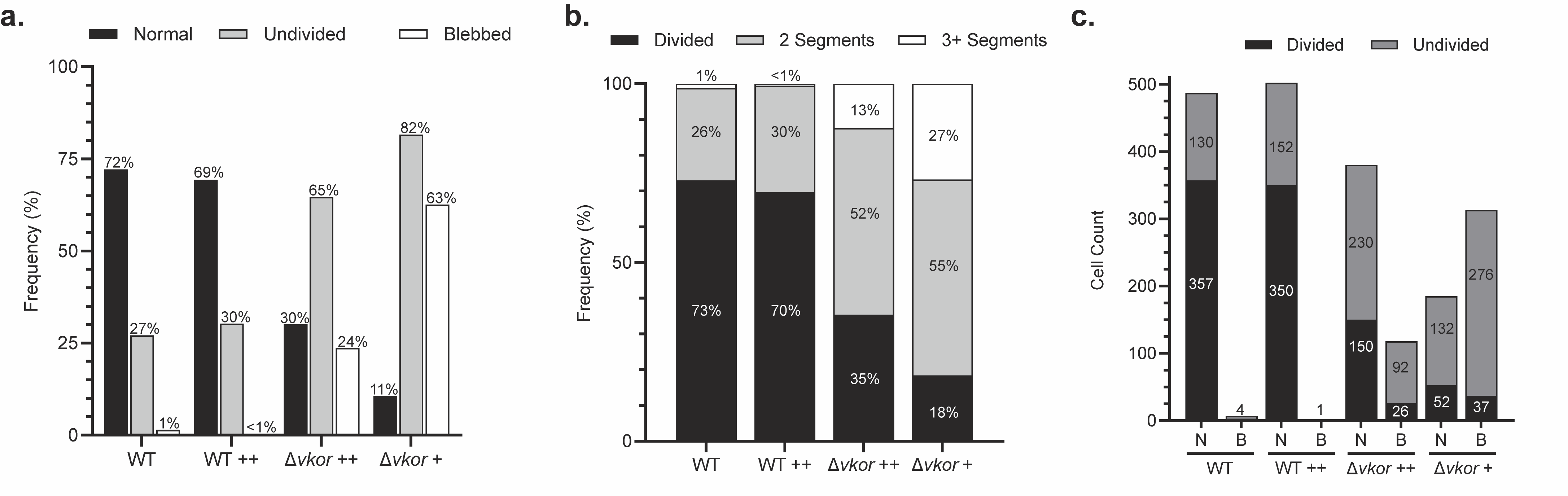
Supplementary Figure 1.** Lack of DSBs in *M. smegmatis* leads to defects in septation and cell division. *M. smegmatis* WT and Δ*vkor* cells, supplemented with 1 mM (++) or 100 µM (+) cystine, were fluorescently stained with 50 nM Syto24 (nucleic acid stain) and 0.6 µg/mL FM4-64 (membrane stain). **a,** Frequencies of each morphology were obtained using FIJI (https://fiji.sc/) by counting cells with bacillar shape, blebbed, and undivided. Undivided was determined by the presence of FM4-64 stained septum. Some cells displayed more than one phenotype leading to total frequencies above 100%. **b,** Undivided cells were categorized into how many septa were present within one filament and frequencies were obtained relative to the total cell count. **c,** Divided and undivided cells displaying normal bacilli (N) or blebbed (B) morphologies were determined. Cell counts included: WT (n=495), WT++ (n=505), Δ*vkor*++ (n=498), Δ*vkor*++ (n=500).

**
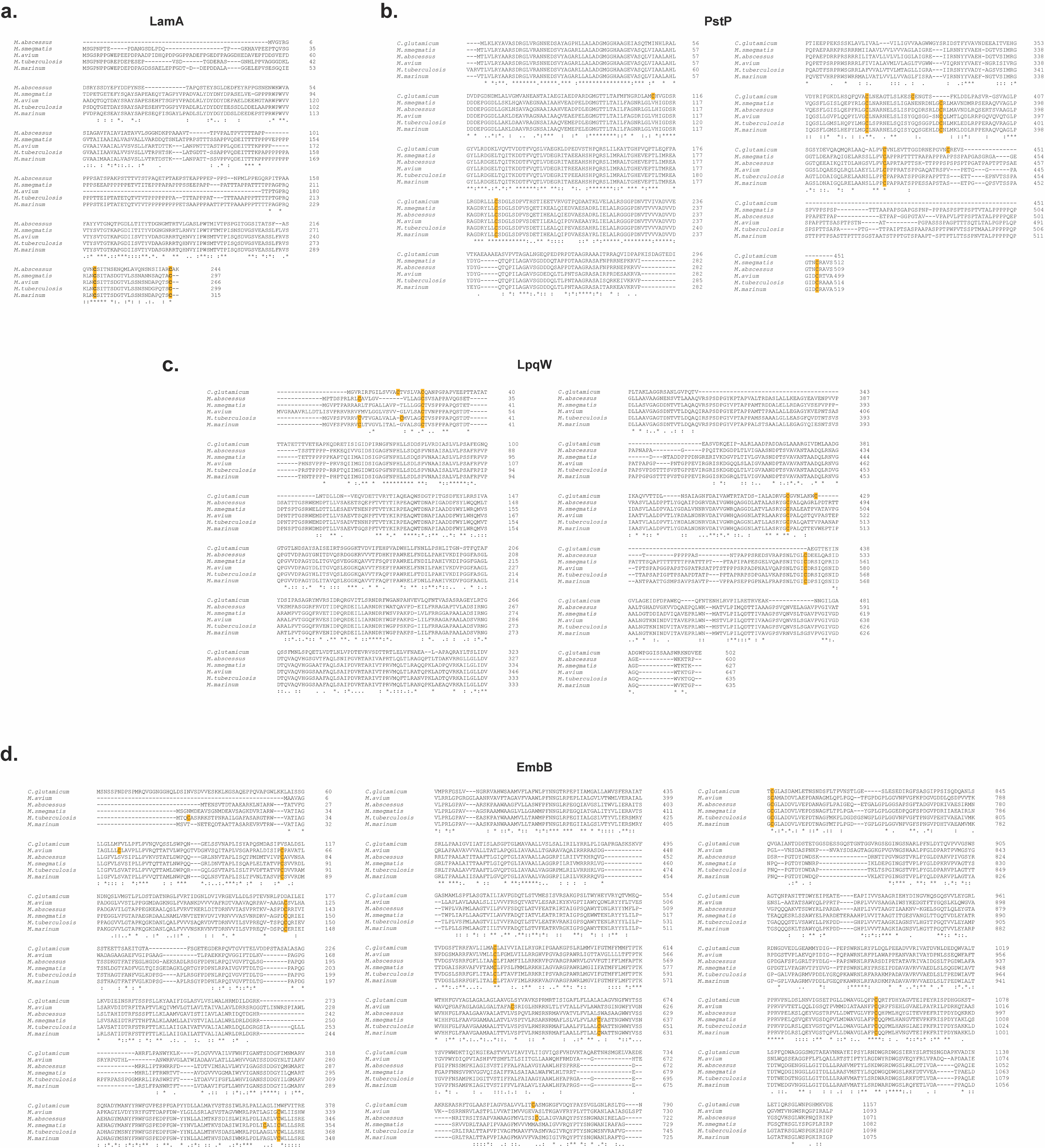
Supplementary Figure 2.** Cysteine conservation in four predicted *M. tuberculosis* DsbA substrates. Protein sequence alignments of LamA (**a**), PstP (**b**), LpqW, (**c**) and EmbB (**d**) were performed using clustal omega^1^. Reference strains used for the protein sequences include *Mycobacterium avium* 104, *Mycobacterium marinum* M, *Mycobacterium smegmatis* mc^2^155, *Mycobacterium tuberculosis* H37Rv, *Mycobacterium abscessus* ATCC 19977, *Corynebacterium glutamicum* strain R. Cysteines are highlighted in yellow.

**
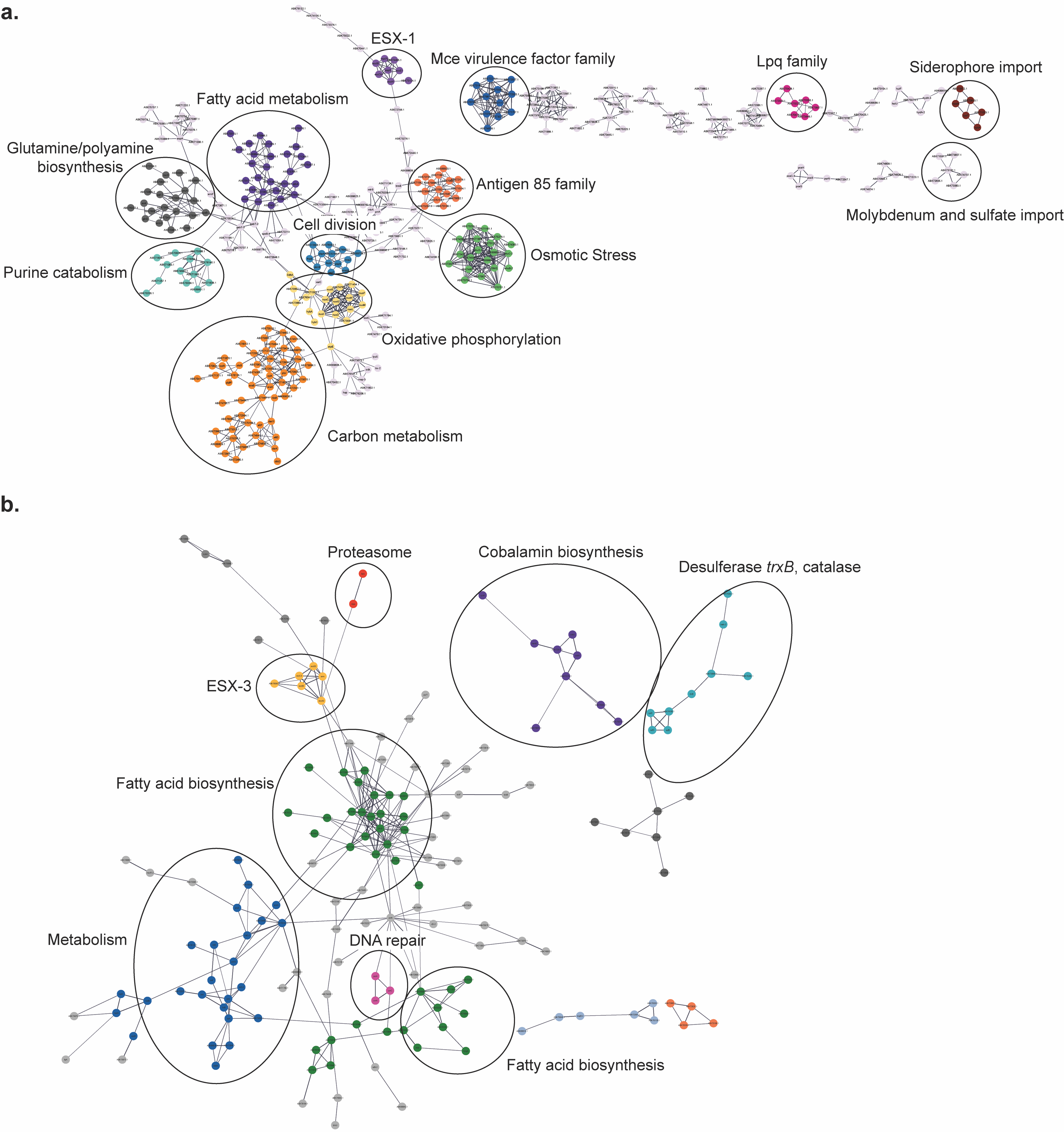
Supplementary Figure 3.** Protein-protein interactions between (**a**) decreased and (**b**) increased proteins found in Δ*vkor*. Predictions were done using STRING DB V12.0. The UniProt ID numbers of increased or decreased proteins found in the whole proteome analysis were uploaded to https://string-db.org/ selecting *M. smegmatis* MC2 155. The string network was set to full and a minimum required interaction score of high confidence (0.7). The network was then exported to Cytoscape 3.10.3 (https://cytoscape.org/) to edit colors and names. Singletons or two protein clusters were not included in the figure.

**
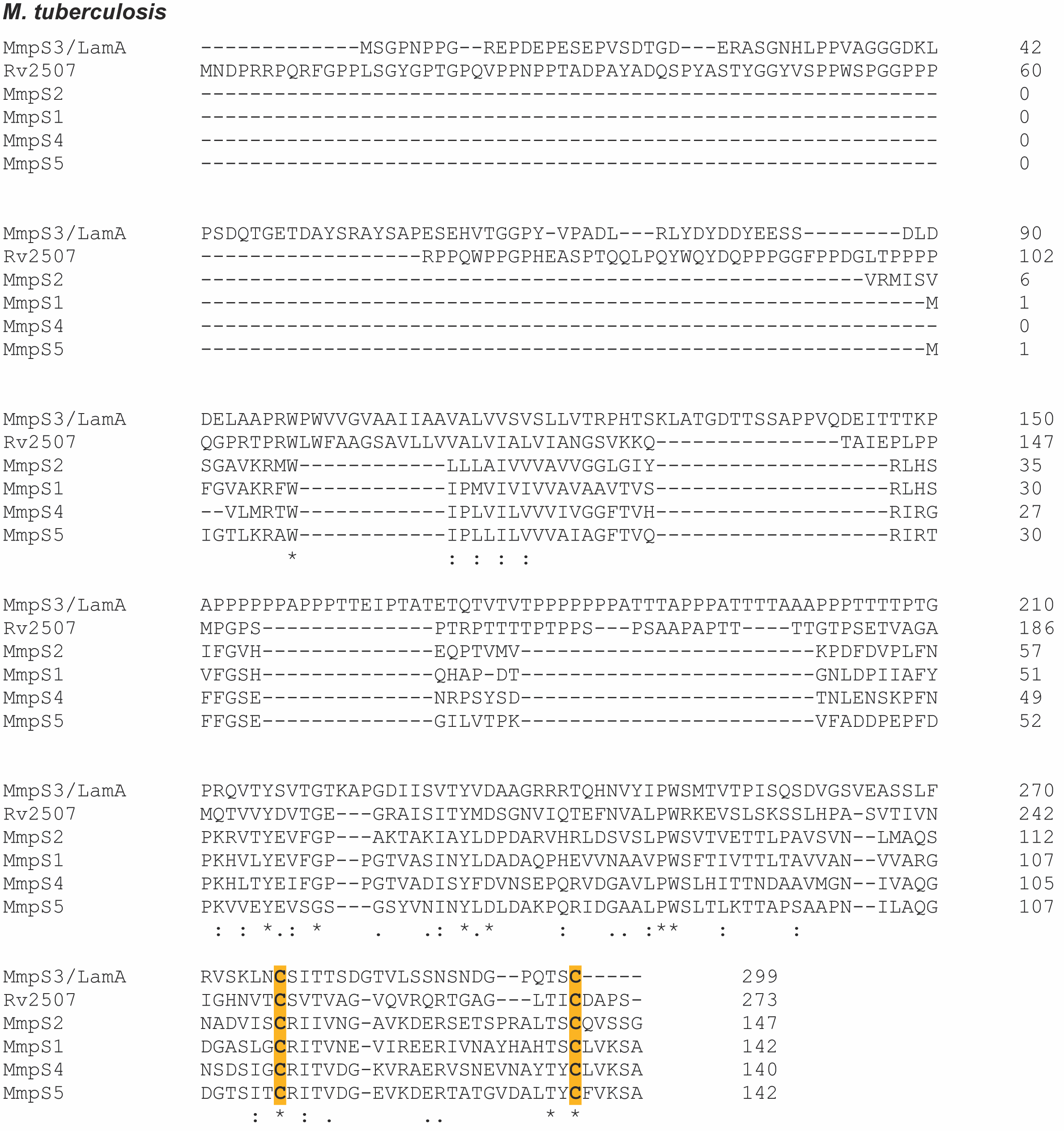
Supplementary Figure 4.** Cysteine conservation among *M. tuberculosis* H37Rv MmpS paralogs. Protein sequence alignments were done using clustal omega^1^. Cysteines are highlighted in yellow.

**
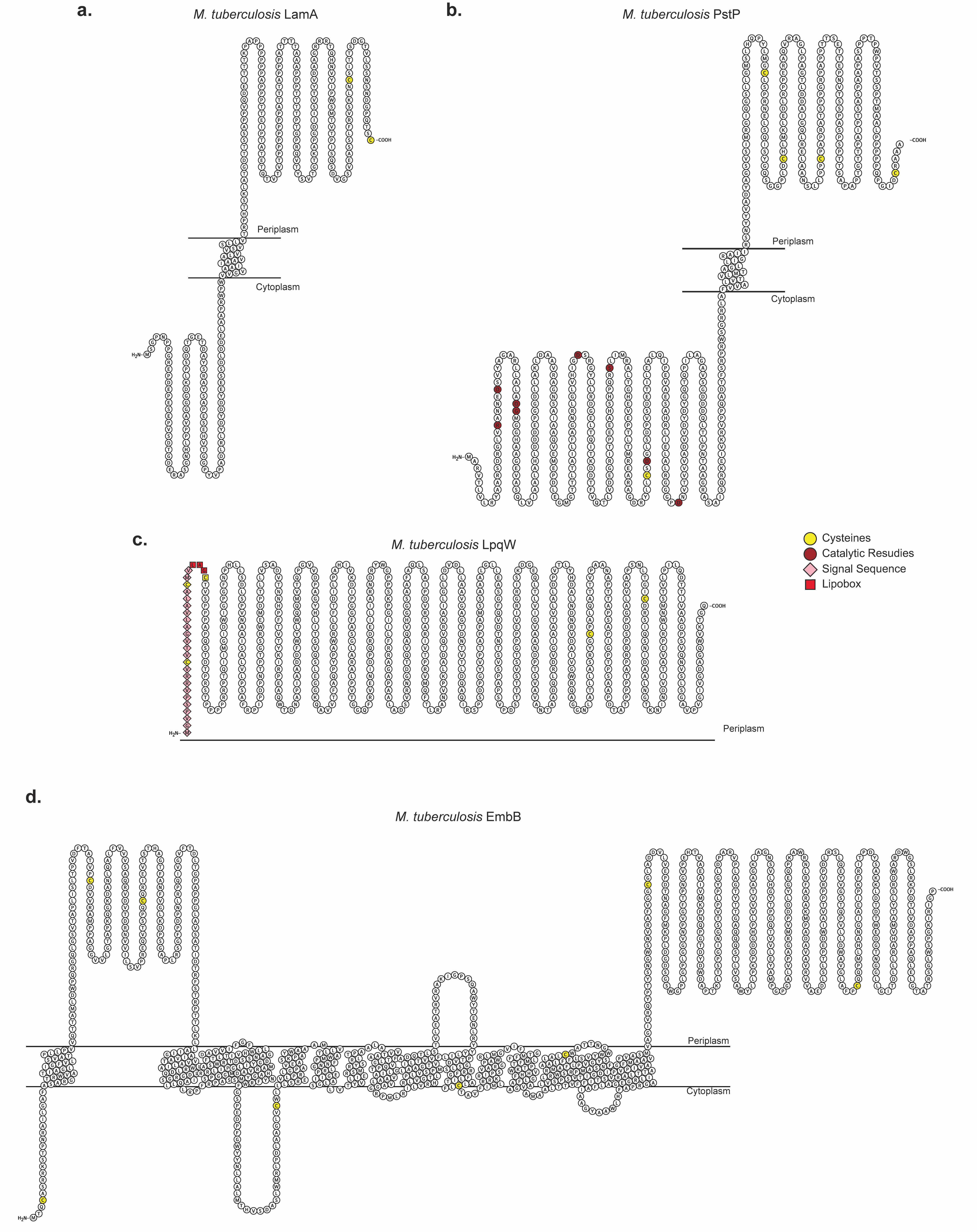
 Supplementary Figure 5.** Membrane protein topologies of four *M. tuberculosis* proteins and the localization of their cysteine residues. Protein topologies of LamA (**a**), PstP (**b**), LpqW (**c**), EmbB (**d**) were visualized using Protter^2^.

**
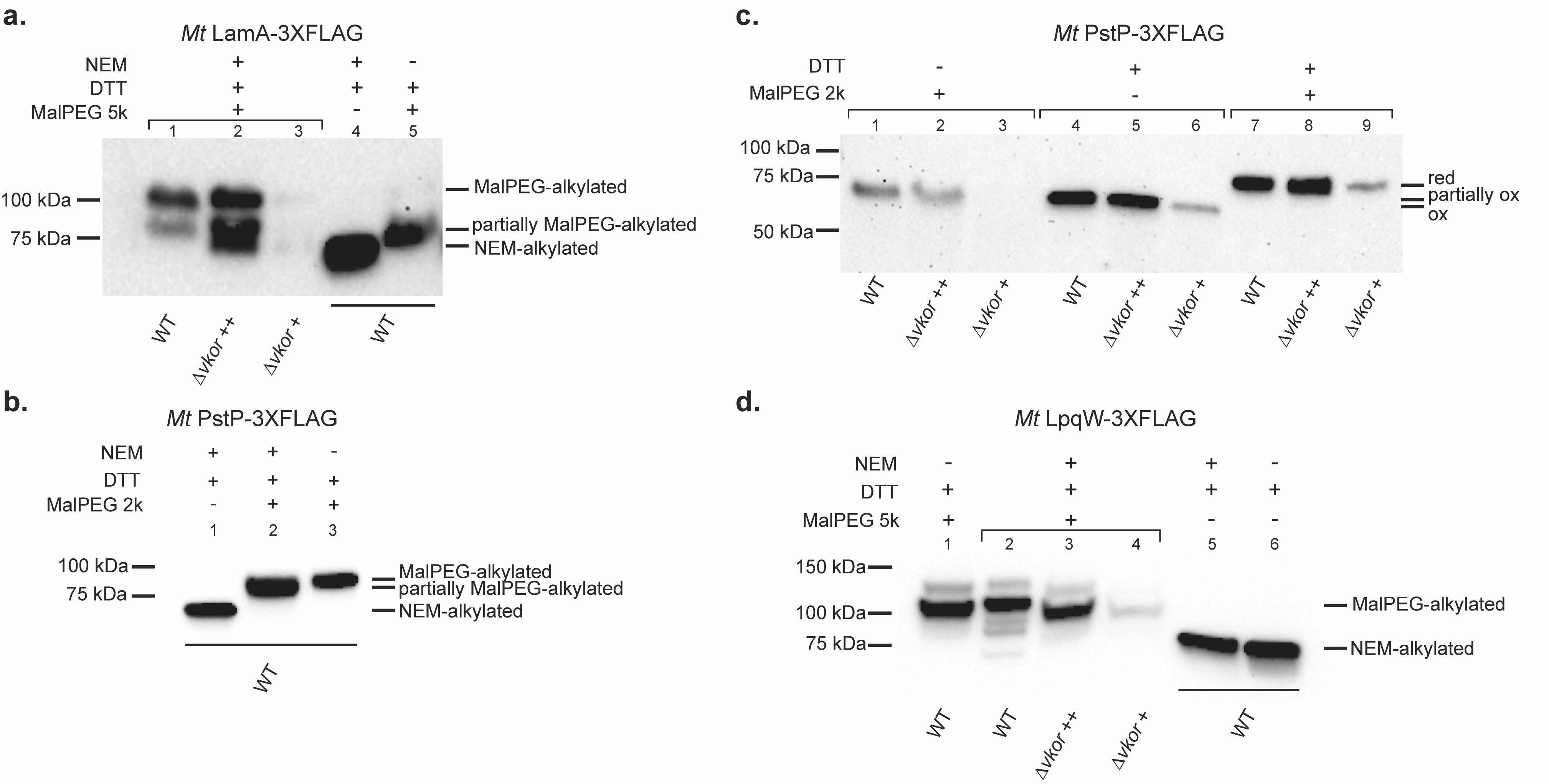
Supplementary Figure 6.** Differential and conventional alkylation corroborate DSBs in three DsbA substrates. *M. tuberculosis* *Mt*LamA (**a**), *Mt*PstP (**b** and **c**), and *Mt*LpqW (**d**) proteins were fused to a 3X-FLAG tag at their carboxy termini and expressed in *M. smegmatis* WT and Δ*vkor* supplemented with 1 mM (++) or 0.4-0.5 mM cystine (+). Cells were grown at 37ºC in the presence of 200 nM aTc for 36 h (PstP and LpqW) or with 5 nM aTc for 18 h (LamA). Proteins were precipitated from cell extracts and differentially alkylated (**a**, **b** and **d**) by treating them with 20 mM NEM to block free thiols. Disulfide-bonded cysteines were then reduced with 100 mM DTT, and new thiols were alkylated with 12.5 mM MalPEG2k or MalPEG5k when indicated. Controls were treated with 100 mM DTT and then alkylated with either 20 mM NEM or 12.5 mM MalPEG2k or MalPEG5k as indicated. Δ*vkor* samples were loaded in excess to be able to observe alkylated bands. Western blotting using α-FLAG antibody was used to detect the proteins. **c,** *In vivo* alkylation of PstP samples (lanes 1-3) were only alkylated with 12.5 mM MalPEG2k. Controls (lanes 4-9) were treated with 100 mM DTT and then alkylated with 12.5 mM MalPEG2k. Immunoblots are representative images of two independent experiments.**
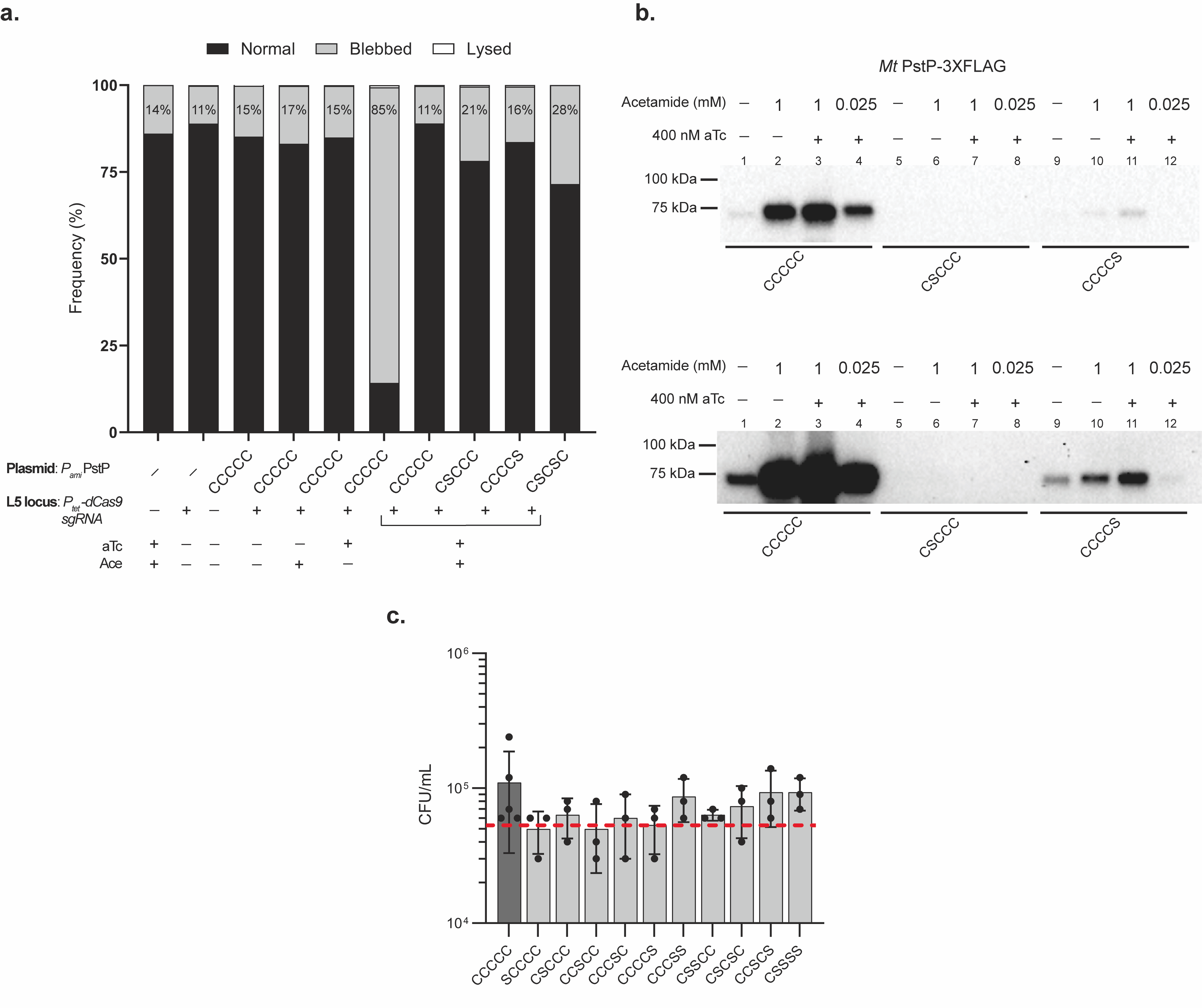
 Supplementary Figure 7.** *M. tuberculosis* PstP cysteine mutants are degraded and display slightly more blebbed events when carrying only one DSB. **a,** *M. smegmatis* *pstP* was silenced using CRISPRi (inducible with aTc), while an ectopic copy of *M. tuberculosis* *pstP*, Cys359Ser or Cys510Ser was used to rescue the knockdown growth. Cells were inoculated to an OD_600_ of 0.01 in 7H9 broth supplemented with 400 nM aTc and/or 25 µM acetamide and incubated at 37ºC for 24 h. Cells were stained and imaged to calculate the frequency of blebbing in 500 cells. **b,** The first DSB between Cys359 and Cys380 of PstP provides more stability than the second DSB between Cys424 and Cys510. Cells were inoculated to an OD_600_ of 0.01 in 7H9 broth supplemented with 400 nM aTc and different concentrations of acetamide. Cells were incubated at 37ºC for 24 h. Proteins were precipitated from cell lysates and reduced with 100 mM DTT. A representative image of two independent experiments is shown. Bottom, overexposed immunoblot to show fainter bands. **c,** CRISPRi silences *M. smegmatis* *pstP* and causes low bacterial titers in strains carrying an ectopic copy of *M. tuberculosis* *pstP* (WT or cysteine mutants) when not induced with acetamide. Cells were inoculated to an OD_600_ of 0.01 (CFU/mL indicated as red dotted line) in 7H9 broth supplemented with 400 nM aTc and incubated at 37ºC for 24 h to enumerate bacteria.

**
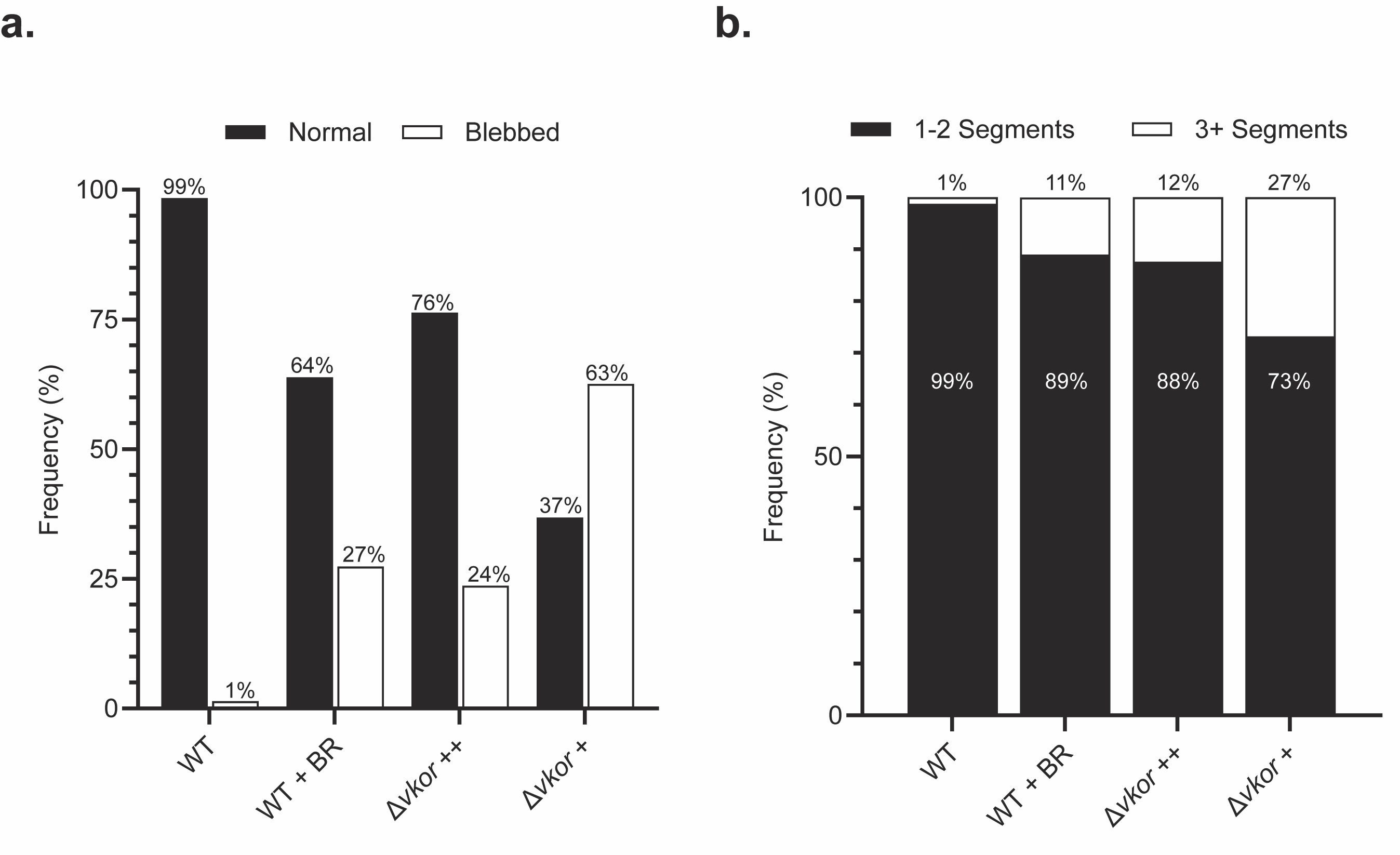
 Supplementary Figure 8.** *M. smegmatis* treatment with a VKOR inhibitor, bromindione (BR), phenocopies the Δ*vkor* morphology grown under high cystine concentration. *M. smegmatis* WT (DMSO control), WT treated with 750 µM of BR, and Δ*vkor* either supplemented with 1 mM (++) or 100 µM (+) cystine, were fluorescently stained with 50 nM Syto24 (nucleic acid stain) and 0.6 µg/mL FM4-64 (membrane stain). **a,** Frequencies of each morphology were obtained using FIJI (https://fiji.sc/) by counting cells with bacillar shape and blebbed. Some cells displayed more than one phenotype leading to total frequencies above 100%. **b,** Undivided cells were categorized into how many septa were present within one filament and frequencies were obtained relative to the total cell count. Cell counts included: WT (n=495), WT+BR (n=490), Δ*vkor*++ (n=498), Δ*vkor*+ (n=500)**.**

**Supplementary Table 1.** Candidate essential substrates of mycobacterial DsbA. *In silico* analysis of 625 essential proteins revealed 19 proteins conserved across five mycobacterial species containing extracytoplasmic cysteine residues thus representing candidate substrates of DsbA-VKOR system. GO class was obtained using the gene ontology resource (https://geneontology.org/). #Cys, indicates cysteine residues present in *M. tuberculosis* H37Rv proteins.

|  | **Gene** | ***M. tuberculosis*** | ***M. smegmatis*** | ***M. marinum*** | ***M. avium*** | ***M. abscessus*** | **# Cys** | **GO Class** |
| --- | --- | --- | --- | --- | --- | --- | --- | --- |
| **Cell Envelope Biogenesis/Maintenance** | ***pstP*** | Rv0018c | MSMEG_0033 | MMAR_0020 | MAV_0022 | MAB_0037c | 5 | Serine/threonine protein phosphatase |
|  | ***culp6*** | Rv3802c | MSMEG_6394 | MMAR_5366 | MAV_0216 | MAB_0178 | 4 | Cutinase activity |
|  | ***pbpB*** | Rv2163c | MSMEG_4233 | MMAR_3200 | MAV_2330 | MAB_2000 | 3 | penicillin binding |
|  | ***murX/***  ***mraY*** | Rv2156c | MSMEG_4230 | MMAR_3196 | MAV_2333 | MAB_2003 | 5 | peptidoglycan biosynthesis |
|  | ***embC*** | Rv3793 | MSMEG_6387 | MMAR_5355 | MAV_0225 | MAB_0189c | 7 | Cell wall biogenesis |
|  | ***embB*** | Rv3795 | MSMEG_6389 | MMAR_5357 | MAV_0225 | MAB_0185c | 8 | Cell wall biogenesis |
|  | ***pks13*** | Rv3800c | MSMEG_6392 | MMAR_5364 | MAV_0218 | MAB_0180 | 5 | Lipid metabolism |
|  | ***aftB*** | Rv3805c | MSMEG_6400 | MMAR_5369 | MAV_0212 | MAB_0174 | 5 | Cell wall biogenesis |
|  | ***aftD*** | Rv0236c | MSMEG_0359 | MMAR_0496 | MAV_4923 | MAB_4472 | 7 | Cell wall biogenesis |
|  | ***lpqW*** | Rv1166 | MSMEG_5130 | MMAR_4288 | MAV_1308 | MAB_1315 | 5 | Phospholipid metabolism |
| **Secretion** | ***lepB*** | Rv2903c | MSMEG_2441 | MMAR_1805 | MAV_3758 | MAB_3223c | 6 | Peptidase activity |
|  | ***eccB3*** | Rv0283 | MSMEG_0616 | MMAR_0542 | MAV_4870 | MAB_2233c | 2 | ATPase activity |
|  | ***mycP3*** | Rv0291 | MSMEG_0624 | MMAR_0550 | MAV_4862 | MAB_2225c | 5 | Protease activity |
|  | ***secY*** | Rv0732 | MSMEG_1483 | MMAR_1070 | MAV_4434 | MAB_3784c | 3 | Translocase activity |
| **Uncharacterized** | **Rv1456c** | Rv1456c | MSMEG_3117 | MMAR_2261 | MAV_3324 | MAB_2754 | 2 | heme biosynthetic process |
|  | **Rv0479c** | Rv0479c | MSMEG_0923 | MMAR_0804 | MAV_4671 | MAB_4077 | 2 | Plasma membrane |
|  | **Rv0526** | Rv0526 | MSMEG_0971 | MMAR_0872 | MAV_4619 | MAB_3976c | 3 | Oxidoreductase activity |
|  | **Rv0528** | Rv0528 | MSMEG_0973 | MMAR_0874 | MAV_4617 | MAB_3974c | 3 | Cytochrome complex assembly |
|  | **Rv2507** | Rv2507 | MSMEG_4720 | MMAR_3854 | MAV_1668 | MAB_1539c | 2 | Membrane |

**Supplementary Table 2.** Proteomics raw data set. List of proteins found in *M. smegmatis* wildtype and Δ*vkor* mutant. Proteins significantly decreased or increased in the Δ*vkor* mutant are indicated with their localization prediction. List of peptides and their modifications through cysteine labeling assay are provided with their localization prediction and essentiality. The table is provided in an Excel document with 6 tabs S2a to S2f.

**Supplementary Table 3.** Essential proteins and their overlap found through *in silico*, global and cysteine enrichment proteomic analyses.

| ***In silico*** | **Proteome wide analysis** | | **Oxidized Cys analysis** | **Essentiality (*M. tuberculosis*)** | ***M tuberculosis* ortholog** |
| --- | --- | --- | --- | --- | --- |
|  | **Decreased** | **Increased** |  |  |  |
|  | A0R565 |  | A0R565 | ES | Probable conserved membrane protein |
|  | A0QUX3 |  | A0QUX3 | ES | LppZ |
| A0QPD4 | A0QPD4 |  | A0QPD4 | ES | AftD |
| A0QNG5 | A0QNG5 |  | A0QNG5 | ESD | PstP |
| A0QQY8 | A0QQY8 |  | A0QQY8 | ES | Rv0479c, uncharacterized |
|  |  | A0QQ40 | A0QQ40 | ES | EccC3 |
|  |  | A0QQ48 | A0QQ48 | ES | EccE3 |
| A0R619 | A0R619 |  |  | ES | Probable conserved membrane protein |
|  | A0R408 |  |  | ES | PrrB |
|  | A0R205 |  |  | ES | AtpE |
| A0R022 | A0R022 |  |  | ESD | PbpB |
|  | A0R017 |  |  | ESD | FtsW |
|  | A0QYQ8 |  |  | ES | hypothetical protein |
|  | A0QNG1 |  |  | ES | PknB |
|  |  | A0R6H8 |  | ESD | IrtA |
|  |  | A0R5D6 |  | ES | Probable conserved transmembrane protein |
|  |  | A0R2A3 |  | ES | Probable acyltransferase |
|  |  | A0QVL8 |  | ES | EfpA |
|  |  | A0QTF1 |  | ES | Probable conserved membrane protein |
|  |  | A0QQ46 |  | ES | EccD3 |
| A0QQ39 |  | A0QQ39 |  | ES | EccB3 |
|  |  |  | A0R2I1 | ES | FdxC |
|  |  |  | A0R0M4 | ES | CtaD |
|  |  |  | A0R030 | ES | MptA |
|  |  |  | A0QWG7 | ESD | Probable conserved membrane protein |
|  |  |  | A0QVT6 | ES | FtsK |
|  |  |  | A0QV12 | ES | DsbA |
|  |  |  | A0QUX0 | ES | GatA |
|  |  |  | A0QTL8 | ES | AroA |
|  |  |  | A0QR37 | ES | Probable conserved membrane protein |
|  |  |  | A0QQB0 | ES | Probable iron-sulfur-binding reductase |
|  |  |  | A0QP27 | ESD | MmpL3 |
|  |  |  | A0R7J1 | ES | Probable peptidoglycan hydrolase |
|  |  |  | A0R7I4 | ES | Hypothetical protein |
| A0R625 |  |  | A0R625 | ES | AftB |
| A0R614 |  |  | A0R614 | ES | EmbB |
|  |  |  | A0R613 | ES | EmbA |
| A0R612 |  |  | A0R612 | ES | EmbC |
| A0R617 |  |  | A0R617 | ES | Pks13 |
| A0QV43 |  |  | A0QV43 | ES | LepB |
| A0QR37 |  |  | A0QR37 | ES | Rv0528, uncharacterized |
| A0R2I8 |  |  |  | ES | LpqW |

ES: Essential

ESD: Essential domain

**Supplementary Table 4.** Distance of Cα-Cα between cysteine (Cys) residues in the AlphaFold predicted structures^3^ of the 42 essential proteins found through *in silico*, global and cysteine enrichment proteomic analyses from Supplementary Table 3. The distances between the Cα atoms for disulfide bonded cysteines range between 3.0 Å and 7.5 Å. Cys location were predicted using TOPCONS^4^ and DeepTMHMM^5^.

| **Protein** | **Uniprot ID** | ***M. smegmatis*** | **Cα-Cα (Å)** | ***M. tuberculosis*** | **Cα-Cα (Å)** |
| --- | --- | --- | --- | --- | --- |
|  | A0R565 | Cys173-Cys205 | 4.52 | Cys111-Cys147 | 4.48 |
|  |  |  |  | Cys174-Cys206 | 4.51 |
| **LppZ** | A0QUX3 | Cys55-Cys64 | 5.21 | Cys64-Cys73 | 5.23 |
|  |  | Cys228-Cys272 | 4.26 | Cys237-Cys281 | 4.26 |
| **AftD** | A0QPD4 | Cys878-Cys887 | 4.81 | Cys886-Cys895 | 4.83 |
|  |  | Cys1096-Cys1131 | 5.58 | Cys1103-Cys1138 | 5.61 |
| **PstP** | A0QNG5 | Cys356-Cys377 | 4.64 | Cys359-Cys380 | 4.65 |
|  |  | Cys421-Cys508 | 4.79 | Cys424-Cys510 | 4.73 |
| **Rv0479c** | A0QQY8 | Cys111-Cys307 | 6.68 | Cys-149-Cys344 | 4.47 |
| **EccC3** | A0QQ40 | 2 Cys (Not exported) | >100 | 2 Cys (Not exported) | >100 |
| **EccE3** | A0QQ48 | 2 Cys (Not exported) | >18 | 3 Cys (Not exported) | >15 |
| **Culp6** | A0R619 | Cys73-Cys165 | 5.14 | Cys72-Cys164 | 5.21 |
|  |  | Cys265-Cys272 | 4.89 | Cys264-Cys271 | 4.79 |
| **PrrB** | A0R408 | 1 Cys (in the membrane) | - | 2 Cys (in the membrane) | >89 |
| **AtpE** | A0R205 | 0 Cys | - | 0 Cys | - |
| **PbpB** | A0R022 | Cys572-Cys574 | 3.64 | Cys602-Cys605 | 3.67 |
| **FtsW** | A0R017 | 0 Cys | - | 1 Cys | - |
|  | A0QYQ8 | 1 Cys (Not exported) | - | 2 Cys (Not exported) | >53 |
| **PknB** | A0QNG1 | Cys499-Cys507 | 4.62 | Cys123-Cys204 | 13.34 |
| **IrtA** | A0R6H8 | 1 Cys (Not exported) | - | 2 Cys (Not exported) | >65 |
|  | A0R5D6 | 2 Cys (Not exported) | >100 | 1 Cys (Not exported) |  |
|  | A0R2A3 | 3 Cys (Not exported) | >10 | 4 Cys (Not exported) | >16 |
| **EfpA** | A0QVL8 | 2 Cys (Not exported) | >22 | 3 Cys (Not exported) | >20 |
|  | A0QTF1 | 0 Cys | - | 1 Cys | - |
| **EccD3** | A0QQ46 | 2 Cys (Not exported) | >23 | 2 Cys (Not exported) | >22 |
| **EccB3** | A0QQ39 | Cys177-Cys376 | 4.7 | Cys181-Cys387 | 4.7 |
| **FdxC** | A0R2I1 | 8 Cys (Iron sulfur cluster) | | | |
| **CtaD** | A0R0M4 | 3 Cys (Not exported) | >26 | 4 Cys (Not exported)  Cys45-48 | 5.47 |
| **MptA** | A0R030 | 4 Cys (Not exported) | >18 | 3 Cys (Not exported) | >19 |
|  | A0QWG7 | 1 Cys (Not exported) | - | 2 Cys (Not exported) | >11 |
| **FtsK** | A0QVT6 | 4 Cys (Not exported) | >22 | 5 Cys (Not exported) | >35 |
| **DsbA** | A0QV12 | Cys86-Cys89 | 4.35 | Cys89-Cys92 | 4.39 |
|  |  | Cys136-Cys188 | 4.82 | Cys140-Cys192 | 4.84 |
| **GatA** | A0QUX0 | 6 Cys (Not exported) | >10 | 7 Cys (Not exported) | >11 |
| **AroA** | A0QTL8 | Cys-102-Cys369 | 31.63 | Cys102-Cys369 | 31.49 |
|  | A0QR37 | Cys228-Cys253 | 4.73 | Cys194-Cys259 | 4.73 |
|  | A0QQB0 | 17 Cys (Iron sulfur cluster) | | | |
| **MmpL3** | A0QP27 | 2 Cys (Not exported) | 41.47 | 4 Cys (3 in the membrane and one not exported) | >7 |
|  | A0R7J1 | 3 Cys | >13 | 4 Cys | >13 |
|  | A0R7I4 | Cys268-Cys324 | 3.85 | Cys273-Cys332 | 3.79 |
| **AftB** | A0R625 | Cys580-Cys625 | 5.19 | Cys568-Cys613 | 5.14 |
| **EmbB** | A0R614 | Cys85-Cys145 | 4.53 | Cys85-Cys145 | 4.48 |
|  |  | Cys731-Cys980 | 4.69 | Cys747-Cys996 | 4.8 |
| **EmbA** | A0R613 | Cys71-Cys131 | 4.71 | Cys76-Cys136 | 4.49 |
|  |  | Cys718-Cys975 | 4.83 | Cys733-Cys989 | 4.69 |
| **EmbC** | A0R612 | Cys74-Cys138 | 4.61 | Cys88-Cys152 | 4.54 |
|  |  | Cys73-Cys973 | 4.57 | Cys749-Cys993 | 4.72 |
| **Pks13** | A0R617 | 6 Cys | >20 | 5 Cys | >20 |
| **LepB** | A0QV43 | Cys90-Cys93 | 3.46 | Cys104-Cys107 | 3.5 |
|  |  | Cys172-Cys204 | 4.87 | Cys186-Cys218 | 4.81 |
|  |  | Cys236-Cys249 | 4.71 | Cys2250-Cys254 | 4.52 |
| **Rv0528** | A0QR37 | Cys228-Cys253 | 4.73 | Cys194-Cys219 | 4.73 |
| **LpqW** | A0R2I8 | Cys490-Cys552 | 4.77 | Cys499-Cys559 | 4.84 |

**Supplementary Table 5.** Summary of substrates of mycobacterial DsbA.

| **Protein** | **Gene Locus** | **Essentiality*** | **# Cys** | **DSBs** | **DB-modified Cys^&^** | **Protein production host** | **Ref.** |
| --- | --- | --- | --- | --- | --- | --- | --- |
| Antigen 85A (FbpA) | Rv3804c | NE | 3 | 1 | NA | *E. coli* | ^6^ |
|  | MSMEG_6398 |  | 4 |  | Cys258, Cys281 | *M. smegmatis* | This study |
| LpqW | Rv1166 | ES | 5 | 1 | NA | *E. coli* | ^7^ |
|  | MSMEG_5130 |  | 3 |  | Cys439 | *M. smegmatis* | This study |
| EspA | Rv3616c | NE | 1 | 1 | NA | *M. tuberculosis* and *M. smegmatis* | ^8^ |
| MycP3 | Rv0291 | ES | 5 | 2 | NA | *E. coli* | ^9^ |
|  | MSMEG_0624 |  |  |  | ND | *M. smegmatis* | This study |
| MmpS5 | Rv0677c | NE | 2 | 1 | NA | *E. coli* | ^10^ |
|  | MSMEG_3495 |  |  |  | Cys153 | *M. smegmatis* | This study |
| EmbC | Rv3793 (C’terminal domain) | ES | 7 | 1 | NA | *E. coli* | ^11^ |
|  | MSMEG_6387 |  | 8 | 1 | NA | *M. smegmatis* | ^12^ |
|  |  |  |  |  | Cys138, Cys973 | *M. smegmatis* | This study |
| Culp6 | Rv3802c | ES | 4 | 2 | NA | *E. coli* | ^13^ |
|  | MSMEG_6394 |  |  |  | ND | *M. smegmatis* | This study |
| MycP1 | Rv3883c | NE | 4 | 2 | NA | *E.coli* | ^14^ |
|  | MSMEG_0083 |  |  |  | Cys51, Cys206, Cys244 | *M. smegmatis* | This study |
| PbpB | Rv2163c | ESD | 3 | 1 | NA | *E. coli* | ^15^ |
|  | MSMEG_4233 |  | 2 |  | ND | *M. smegmatis* | This study |
| AftB | MSMEG_6400 | ES | 4 | 1 | Cys357, Cys580, Cys625 | *M. smegmatis* | This study |
| AftD | MAB_4472 | ES | 9 | 2 | NA | *E. coli* | ^16^ |
|  | MSMEG_0359 |  | 7 |  | Cys887, Cys878 | *M. smegmatis* | This study |
| EccB3 | MSMEG_0616 | ES | 3 | 1 | NA | *M. smegmatis* | ^17^ |
|  |  |  |  |  | ND | *M. smegmatis* | This study |
| LamA (MmpS3) | Rv2198c | NE | 2 | 1 | NA | *M. smegmatis* | This study |
|  | MSMEG_4265 |  |  |  | ND |  |  |
| PstP | Rv0018c | ESD | 5 | 2 | NA | *M. smegmatis* | This study |
|  | MSMEG_0033 |  |  |  | Cys186, Cys356, Cys377 |  |  |
| EmbB | Rv3795 | ES | 8 | 2 | NA | *M. smegmatis* | This study |
|  | MSMEG_6389 |  |  |  | Cys85, Cys145 |  | This study and ^12^ |

***** NE: Not essential, ES: Essential, ESD: Essential domain^18^.

**^&^** Oxidized cysteine modified with DBIA and identified by mass spectrometry.

ND: Peptides were not detected

NA: Not applicable

**Supplementary Table 6.** List of strains and plasmids used in this study.

| **ID** | **Genotype** | **Ref** |
| --- | --- | --- |
| *Strains* | | |
| NK168 | *M. smegmatis* mc^2^155 | E. Rubin Lab |
| RD149 | *M. smegmatis* mc^2^155 Δ*vkor* | ^19^ |
| NK317 | *M. smegmatis* mc^2^155 Δ*dsbA*::pTetG-*MsdsbA* | ^20^ |
| FLAG-tagged strains | | |
| LL191 | *M. smegmatis* mc^2^155 pTetG-*MtmmpS3*-3xFLAG | This study |
| LL196 | *M. smegmatis* mc^2^155 Δvkor pTetG-*MtmmpS3*-3xFLAG | This study |
| LL148 | *M. smegmatis* mc^2^155 pTetG-*MtpstP*-3xFLAG | This study |
| LL163 | *M. smegmatis* mc^2^155 Δ*vkor* pTetG-*MtpstP*-3xFLAG | This study |
| LL343 | *M. smegmatis* mc^2^155 pTetG-*MtlpqW*-3xFLAG | This study |
| LL344 | *M. smegmatis* mc^2^155 Δvkor pTetG-*MtlpqW*-3xFLAG | This study |
| LL419 | *M. smegmatis* mc^2^155 pTetG-*MtembB*-3xFLAG | This study |
| LL420 | *M. smegmatis* mc^2^155 Δvkor pTetG-*MtembB*-3xFLAG | This study |
| LL189 | *M. smegmatis* mc^2^155 pTetG-*MtmurF*-3xFLAG | This study |
| LL194 | *M. smegmatis* mc^2^155 Δ*vkor* pTetG-*MtmurF*-3xFLAG | This study |
| MtPstP folding mutants | | |
| LL398 | *M. smegmatis* mc^2^155 pTetG-*Mt*pstP_C510S_-3XFLAG | This study |
| LL399 | *M. smegmatis* mc^2^155 pTetG-*Mt*pstP_C510S, C424S_-3XFLAG | This study |
| LL400 | *M. smegmatis* mc^2^155 pTetG-*Mt*pstP_C510S, C424S, C380S_-3XFLAG | This study |
| LL401 | *M. smegmatis* mc^2^155 pTetG-*Mt*pstP_C510S, C424S, C380S, C359S_-3XFLAG | This study |
| LL402 | *M. smegmatis* mc^2^155 pTetG-*Mt*pstP_C510S, C424S, C380S, C359S, C189S_-3XFLAG | This study |
| LL423 | *M. smegmatis* mc^2^155 pTetG-*Mt*pstP_C359S_-3XFLAG | This study |
| LL424 | *M. smegmatis* mc^2^155 pTetG-*Mt*pstP_C380S_-3XFLAG | This study |
| LL468 | *M. smegmatis* mc^2^155 pTetG-*Mt*pstP_C359S, C380S_-3XFLAG | This study |
| LL469 | *M. smegmatis* mc^2^155 pTetG-*Mt*pstP_C359S, C424S_-3XFLAG | This study |
| LL470 | *M. smegmatis* mc^2^155 pTetG-*Mt*pstP_C380S, C510S_-3XFLAG | This study |
| LL471 | *M. smegmatis* mc^2^155 pTetG-*Mt*pstP_C189S_-3XFLAG | This study |
| MsPstP knockdown and cysteine mutants | | |
| LL464 | *M. smegmatis* mc^2^155 L5::P*_tet_-*Sth1-*MspstP* sgRNA, P_tet_-Sth1 dCas9 | This study |
| LL466 | *M. smegmatis* mc^2^155 L5::P*_tet_-*Sth1-*MspstP* sgRNA, P_tet_-Sth1 dCas9, P*ami*-*MtpstP*-3XFLAG | This study |
| LL514 | *M. smegmatis* mc^2^155, L5::P*_tet_-*Sth1-*MspstP* sgRNA, P_tet_-Sth1 dCas9, P*ami*-*MtpstP*_C189S_-3XFLAG | This study |
| LL515 | *M. smegmatis* mc^2^155 L5::P*_tet_-*Sth1-*MspstP* sgRNA, P_tet_-Sth1 dCas9, P*ami*-*MtpstP*_C359S_-3XFLAG | This study |
| LL516 | *M. smegmatis* mc^2^155 L5::P*_tet_-*Sth1-*MspstP* sgRNA, P_tet_-Sth1 dCas9, P*ami*-*MtpstP*_C380S_-3XFLAG | This study |
| LL517 | *M. smegmatis* mc^2^155 L5::P*_tet_-*Sth1-*MspstP* sgRNA, P_tet_-Sth1 dCas9, P*ami*-*MtpstP*_C424S_-3XFLAG | This study |
| LL518 | *M. smegmatis* mc^2^155 L5::P*_tet_-*Sth1-*MspstP* sgRNA, P_tet_-Sth1 dCas9, P*ami*-*MtpstP*_C510S_-3XFLAG | This study |
| LL519 | *M. smegmatis* mc^2^155 L5::P*_tet_-*Sth1-*MspstP* sgRNA, P_tet_-Sth1 dCas9, P*ami*-*MtpstP*_C424S, C510S_-3XFLAG | This study |
| LL525 | *M. smegmatis* mc^2^155 L5::P*_tet_-*Sth1-*MspstP* sgRNA, P_tet_-Sth1 dCas9, P*ami*-*MtpstP*_C359S, C380S_-3XFLAG | This study |
| LL526 | *M. smegmatis* mc^2^155 L5::P*_tet_-*Sth1-*MspstP* sgRNA, P_tet_-Sth1 dCas9, P*ami*-*MtpstP*_C359S, C424S_-3XFLAG | This study |
| LL527 | *M. smegmatis* mc^2^155 L5::P*_tet_-*Sth1-*MspstP* sgRNA, P_tet_-Sth1 dCas9, P*ami*-*MtpstP*_C380S, C510S_-3XFLAG | This study |
| LL528 | *M. smegmatis* mc^2^155 L5::P*_tet_-*Sth1-*MspstP* sgRNA, P_tet_-Sth1 dCas9, P*ami*-*MtpstP*_C359S, C380S, C424S, C510S_-3XFLAG | This study |
| *Plasmids* | | |
| pTetG | Mycobacterial Tet expression vector oriM, colE1 ori, (Hyg^r^) | ^21^ |
| pJR962 | Plasmid for CRISPRi transcriptional repression P*_tet_-*Sth1-sgRNA scaffold, P*_tet_*-Sth1 dCas9, TetR, L5-integrase (Km^r^) | ^22^ |
| pJV126 | Recombineering plasmid with P_hsp60_-*sacB* and acetamidase promoter (Km^r^) | J.van Kessel |
| PL146 | pTetG-*MtmurF-*3XFLAG (Hyg^r^) | This study |
| PL148 | pTetG-*MtlamA*-3XFLAG (Hyg^r^) | This study |
| PL105 | pTetG-*MtpstP-*3XFLAG (Hyg^r^) | This study |
| PL220 | pTetG-*MtlpqW*-3XFLAG (Hyg^r^) | This study |
| PL265 | pTetG-*MtembB*-3XFLAG (Hyg^r^) | This study |
| PL209 | pTetG-*MtpstP*_C510S_-3XFLAG (Hyg^r^) | This study |
| PL243 | pTetG-*MtpstP*_C424S, C510S_-3XFLAG (Hyg^r^) | This study |
| PL249 | pTetG-*MtpstP*_C380S, C424S, C510S_-3XFLAG (Hyg^r^) | This study |
| PL252 | pTetG-*MtpstP*_C359S, C380S, C424S, C510S_-3XFLAG (Hyg^r^) | This study |
| PL258 | pTetG-*MtpstP*_C189S, C359S, C380S, C424S, C510S_-3XFLAG (Hyg^r^) | This study |
| PL275 | pTetG-*MtpstP*_C359S_-3XFLAG (Hyg^r^) | This study |
| PL276 | pTetG-*MtpstP*_C380S_-3XFLAG (Hyg^r^) | This study |
| PL288 | pTetG-*MtpstP*_C359S, C380S_-3XFLAG (Hyg^r^) | This study |
| PL289 | pTetG-*MtpstP*_C359S, C424S_-3XFLAG (Hyg^r^) | This study |
| PL290 | pTetG-*MtpstP*_C380S, C510S_-3XFLAG (Hyg^r^) | This study |
| PL291 | pTetG-*MtpstP*_C189S_-3XFLAG (Hyg^r^) | This study |
| PL282 | pJR962 with *MspstP* (MSMEG_0033) sgRNA (Km^r^) | This study |
| PL283 | pTetG Δ*tetR* (Hyg^R^) | This study |
| PL286 | pTetG Δ*tetR* *amiCA* P*_ami_*-*MtpstP-*3XFLAG (Hyg^r^) | This study |
| PL298 | pTetG Δ*tetR* *amiCA* P*_ami_*-*MtpstP*_C189S_*-*3XFLAG (Hyg^r^) | This study |
| PL299 | pTetG Δ*tetR* *amiCA* P*_ami_*-*MtpstP*_C359S_*-*3XFLAG (Hyg^r^) | This study |
| PL300 | pTetG Δ*tetR* *amiCA* P*_ami_*-*MtpstP*_C380S_*-*3XFLAG (Hyg^r^) | This study |
| PL301 | pTetG Δ*tetR* *amiCA* P*_ami_*-*MtpstP*_C424S_*-*3XFLAG (Hyg^r^) | This study |
| PL302 | pTetG Δ*tetR* *amiCA* P*_ami_*-*MtpstP*_C510S_*-*3XFLAG (Hyg^r^) | This study |
| PL303 | pTetG Δ*tetR* *amiCA* P*_ami_*-*MtpstP*_C424S, C510S_*-*3XFLAG (Hyg^r^) | This study |
| PL304 | pTetG Δ*tetR* *amiCA* P*_ami_*-*MtpstP*_C359S, C380S_*-*3XFLAG (Hyg^r^) | This study |
| PL305 | pTetG Δ*tetR* *amiCA* P*_ami_*-*MtpstP*_C359S, C424S_*-*3XFLAG (Hyg^r^) | This study |
| PL306 | pTetG Δ*tetR* *amiCA* P*_ami_*-*MtpstP*_C380S, C510S_*-*3XFLAG (Hyg^r^) | This study |
| PL307 | pTetG Δ*tetR* *amiCA* P*_ami_*-*MtpstP*_C359S, C380S, C424S, C510S_*-*3XFLAG (Hyg^r^) | This study |

**Supplementary Table 7.** List of primers used in this study.

| **ID** | **Sequence 5’-3’** |
| --- | --- |
| PR151 | ggcagcgactacaaagac |
| PR154 | tggtctttgtagtcgctgcctgccgccgcccggcagtc |
| PR172 | cgaggtcgacggtatcgat |
| PR173 | atgtatatctccttcttaattaagcatg |
| PR174 | attaagaaggagatatacatgtggcgcgcgtgaccctgg |
| PR222 | cctgatgggctccctcagcccgc |
| PR223 | taaggctggtgcagggacatgc |
| PR224 | ccctctcgactcccatctgatgaaac |
| PR225 | cccccagactgtccgtag |
| PR226 | gctgccgccttccccggcgccgc |
| PR227 | agggagttggccgccagttcgcgc |
| PR229 | ggctgaggcgggggtggcg |
| PR230 | gtgtacgaccagcacggc |
| PR231 | attaagaaggagatatacatatgagcgggccgaatccc |
| PR249 | tggtctttgtagtcgctgccgcagctcgtttgcggtcc |
| PR278 | gggcatcgactcccgggcggcggcacac |
| PR289 | ttacctgctgtcctcggacgggttgtc |
| PR290 | cgatcaccggcgcgggct |
| PR291 | gctcacatgttctttcctgcgttatcc |
| PR312 | tggtctttgtagtcgctgcctggaccaattcggatcttgcccggtg |
| PR330 | attaagaaggagatatacatatgacacagtgcgcgagcagacgc |
| PR372 | attaagaaggagatatacatatgggcgtgcccagccca |
| PR373 | tggtctttgtagtcgctgccttgcccggtcttcacccattgg |
| PR472 | tcacatgttctttcctgcgaagtgacgcggtctcaag |
| PR473 | ctccttcttaattaagcatgggtcacccctttccattc |
| PR474 | catgcttaattaagaaggagatatac |
| PR475 | acgcaggaaagaacatgtg |
| PR476 | ccatgggctagcggcttt |
| PR477 | gttgcggagccatctagc |
| PR478 | ccaccgctgttctaacgc |
| PR479 | ccagcaacgcggcctt |
| PR480 | gccgcgtatcgcaggac |
| PR493 | tggtctttgtagtcgctgcctgccgccgcccgggagtc |
| PR496 | gggaggtcgctgcgggccgcgtagc |
| PR497 | aaacgctacgcggcccgcagcgacc |
| PR499 | ggcgttcacccttgactt |

**References**

1. Sievers, F. *et al.* Fast, scalable generation of high-quality protein multiple sequence alignments using Clustal Omega. *Mol Syst Biol* **7**, (2011).

2. Omasits, U., Ahrens, C. H., Mu, S. & Wollscheid, B. Sequence analysis Protter : interactive protein feature visualization and integration with experimental proteomic data. *Bioinformatics* **30**, 884–886 (2014).

3. Jumper, J. *et al.* Highly accurate protein structure prediction with AlphaFold. *Nature* **596**, 583–589 (2021).

4. Bernsel, A., Viklund, H., Hennerdal, A. & Elofsson, A. TOPCONS: Consensus prediction of membrane protein topology. *Nucleic Acids Res* **37**, 465–468 (2009).

5. Hallgren, J. *et al.* DeepTMHMM predicts alpha and beta transmembrane proteins using deep neural networks. *BioRxiv* (2022).

6. Ronning, D. M., Vissa, V., Besra, G. S., Belisle, J. T. & Sacchettini, J. C. Mycobacterium tuberculosis antigen 85A and 85C structures confirm binding orientation and conserved substrate specificity. *Journal of Biological Chemistry* **279**, 36771–36777 (2004).

7. Marland, Z. *et al.* Hijacking of a Substrate-binding Protein Scaffold for use in Mycobacterial Cell Wall Biosynthesis. *J Mol Biol* **359**, 983–997 (2006).

8. Garces, A. *et al.* EspA acts as a critical mediator of ESX1-dependent virulence in Mycobacterium tuberculosis by affecting bacterial cell wall integrity. *PLoS Pathog* **6**, (2010).

9. Wagner, J. M. *et al.* Understanding specificity of the mycosin proteases in ESX/type VII secretion by structural and functional analysis. *J Struct Biol* **184**, 115–128 (2013).

10. Cuthbert, B. J. *et al.* The structure of Mycobacterium thermoresistibile MmpS5 reveals a conserved disulfide bond across mycobacteria. *Metallomics* **16**, (2024).

11. Alderwick, L. J. *et al.* The C-terminal domain of the arabinosyltransferase mycobacterium tuberculosis EmbC is a lectin-like carbohydrate binding module. *PLoS Pathog* **7**, (2011).

12. Zhang, L. *et al.* Structures of cell wall arabinosyltransferases with the anti-tuberculosis drug ethambutol. *Science (1979)* **368**, 1211–1219 (2020).

13. Crellin, P. K. *et al.* Tetrahydrolipstatin inhibition, functional analyses, and three-dimensional structure of a lipase essential for mycobacterial viability. *Journal of Biological Chemistry* **285**, 30050–30060 (2010).

14. Solomonson, M. *et al.* Structure of the mycosin-1 protease from the mycobacterial ESX-1 protein type VII secretion system. *Journal of Biological Chemistry* **288**, 17782–17790 (2013).

15. Lu, Z. *et al.* Structures of Mycobacterium tuberculosis Penicillin-Binding Protein 3 in Complex with Five β-Lactam Antibiotics Reveal Mechanism of Inactivation. *Mol Pharmacol* **97**, 287–294 (2020).

16. Tan, Y. Z. *et al.* Cryo-EM Structures and Regulation of Arabinofuranosyltransferase AftD from Mycobacteria. *Mol Cell* **78**, 683-699.e11 (2020).

17. Famelis, N. *et al.* Architecture of the mycobacterial type VII secretion system. *Nature* **576**, 321–325 (2019).

18. Dejesus, M. A. *et al.* Comprehensive essentiality analysis of the Mycobacterium tuberculosis genome via saturating transposon mutagenesis. *mBio* **8**, (2017).

19. Dutton, R. J. *et al.* Inhibition of bacterial disulfide bond formation by the anticoagulant warfarin. *Proc Natl Acad Sci U S A* **107**, 297–301 (2010).

20. Ke, N. *et al.* Identification of the thioredoxin partner of VKOR in mycobacterial disulfide bond formation. *J Bacteriol* (2018) doi:10.1128/jb.00137-18.

21. Ehrt, S. *et al.* Controlling gene expression in mycobacteria with anhydrotetracycline and Tet repressor. *Nucleic Acids Res* **33**, 1–11 (2005).

22. Rock, J. M. *et al.* Programmable transcriptional repression in mycobacteria using an orthogonal CRISPR interference platform. *Nat Microbiol* **2**, 1–9 (2017).
